# Estimates and projections of the global economic cost of breast cancers from 2021 to 2050

**DOI:** 10.3389/fendo.2025.1692619

**Published:** 2025-12-04

**Authors:** Yun Mao, Xuelei Chu, Feiyu Xie, Ling Fu, Zhixun Ding, Wanhui Zhang, Qisheng Zhang, Chuanen Tang, Shijie Zhu, Wen Cao, Beier Hu

**Affiliations:** 1 Department of Oncology, The Second Affiliated Hospital of Hunan University of Chinese Medicine, Changsha, Hunan, China; 2 Department of Oncology, Guang’anmen Hospital, China Academy of Chinese Medical Sciences, Beijing, China; 3 Integrated Traditional Chinese and Western Medicine Department, The Cancer Hospital of the University of Chinese Academy of Sciences, Zhejiang Cancer Hospital, Hangzhou, China; 4 Department of Spleen and Stomach Diseases, Changping District Traditional Chinese Medicine Hospital, Beijing, China; 5 Department of Traditional Chinese Medicine, Shangyu People’s Hospital, Shaoxing, China; 6 Department of Oncology, Wangjing Hospital, China Academy of Chinese Medical Sciences, Beijing, China

**Keywords:** breast cancer, health disparities, global economic cost, value of lost welfare, global health equity

## Abstract

**Objective:**

To quantify the global macroeconomic burden of breast cancer in 2021, reveal disparities in economic losses across regions, age groups, and gender groups, and provide a basis for optimizing the allocation of prevention and control resources.

**Methods:**

Based on breast cancer disability-adjusted life years (DALY) data from the Global Burden of Disease (GBD 2021) database, combined with World Bank economic indicators, the value of lost welfare (VLW) model was employed to assess economic losses. The model standardized losses across countries using the statistical value of life (VSL), with a core parameter income elasticity of 1.0. Regional disparities were analyzed through stratification by Socio-demographic Index (SDI), and statistical uncertainty was calculated using a Bayesian model with 95% uncertainty intervals.

**Results:**

In 2021, the global VLW due to breast cancer reached 2,538.849 billion US dollars, accounting for 1.65% of global GDP. Regional analysis revealed significant inequality: High-income North America bore the highest economic loss (557.9 billion US dollars), followed by Western Europe (551.4 billion US dollars), while Central Europe, Eastern Europe, and Central Asia, despite lower absolute losses, had an economic burden as a proportion of GDP as high as 3.01%, far exceeding the global average. In terms of population distribution, the female group dominated absolutely (accounting for 24% of female cancer deaths), and economic losses for patients aged 50 and above accounted for over 70%, highlighting the characteristic burden of disease aging. The forecast indicates that the total VLW caused by breast cancer worldwide in 2050 will reach US$21,008.2 billion, with the top regions incurring the highest economic losses being South Asia, East Asia, High-income North America, and Southeast Asia.

**Conclusion:**

Breast cancer causes significant macroeconomic losses and exhibits marked regional inequality, with high-income countries bearing the highest absolute burden, while low- and middle-income regions face more severe relative economic impacts. It is imperative to implement targeted prevention and control strategies based on SDI stratification to promote optimal allocation of global health resources.

## Highlights

The burden of breast cancer profoundly reflects global health inequities: high-SDI countries have reduced mortality rates through advantages in screening and treatment, but at high costs; low- and middle-SDI countries face the fastest-growing burden and economic disasters due to Westernization of risk factors, scarcity of medical resources, and a trend of younger onset. Projections for 2050 show that South Asia and East Asia will become new hotspots, with the burden in China and India surpassing that of the United States. Solutions require global collaboration to tailor screening, treatment, and economic policies according to SDI differences. The evidence base (such as GBD and GLOBOCAN) provides support for policies, but it is necessary to note data limitations, especially in low-SDI countries or regions.

## 1 Introduction

Breast cancer, as the most common malignant tumor in women, accounted for 2.3 million new cases globally in 2022, representing 25% of all female cancers (1). According to the American Cancer Society, the average lifetime risk of breast cancer for women in the United States is approximately 13%, meaning 1 in 8 women will be diagnosed with breast cancer, and about 1 in 43 women (approximately 2%) will die from it (2). In China, data from the National Cancer Center show that in 2022, there were approximately 350,000 new breast cancer cases and 75,000 deaths among Chinese women, accounting for 15.59% and 7.94% of total new cancer cases and deaths, respectively, making it one of the leading causes of cancer-related deaths among women (3). It is projected that by 2050, global breast cancer cases will increase by 38%, and deaths may rise by 68% (4). The World Health Organization (WHO) launched the Global Breast Cancer Initiative, with a core goal to reduce global breast cancer mortality by 2.5% annually and save 2.5 million lives by 2040 (5). The occurrence of breast cancer is associated with immune dysregulation and mutations in oncogenes (6, 7); it is also the result of everyday factors such as high BMI, alcohol consumption, and the intake of red meat (8). Cancer reduces productivity among affected populations and society, leading to unemployment, labor loss, and reduced capital investment, resulting in economic costs. The estimated global economic cost of cancer from 2020 to 2050 is $25.2 trillion (9). In a study of 5,915 breast cancer patients, 34% experienced moderate-to-severe financial hardship (10). In the United States, for triple-negative breast cancer, patients with metastatic recurrence incurred $8,575/month higher medical costs than non-recurrent patients, while those with locoregional recurrence had $3,609/month higher costs (11).

The Global Burden of Disease (GBD) database, led by the Institute for Health Metrics and Evaluation (IHME), covers 204 countries and regions, which are further divided into 21 regions and 7 super-regions. This database compiles extensive data from 1990 to 2021 for these 204 countries and regions through sources such as censuses, household surveys, death registries, and cancer registries, including disease incidence, mortality, disability-adjusted life years (DALYs), and risk-factor exposure, achieving comprehensive coverage across nations and over time. GBD integrates multiple data sources, including vital registration of mortality and verbal autopsy systems, population-based cancer registries, household surveys, hospital and insurance claims, and the national census—subjected to standardized quality checks and the construction of a unified cause-of-death list (12, 13). In the 2021 study, GBD collaborators and this research team assessed the burden of breast cancer across different global regions. Despite extensive efforts, evaluations concerning the macroeconomic impact of breast cancer on regional and/or national economies remain relatively scarce. In fact, to the best of the authors’ knowledge, no studies have yet assessed the macroeconomic consequences of breast cancer globally in a standardized manner. This study aims to fill the gap by evaluating the global disease burden of breast cancer from epidemiological and economic perspectives, analyzing regional disparities caused by age, sex, and geographic factors, to promote rational resource allocation and thereby improve prevention and treatment for breast cancer patients worldwide.

The Value of Lost Welfare (VLW) is a standardized model for assessing the macroeconomic losses caused by disease burden by DALYs and the Value of Statistical Life (VSL) (14, 15). Here, VSL is defined as the amount an individual is willing to pay to reduce the risk of mortality, reflecting the individual’s marginal rate of substitution between wealth and mortality risk (the trade-off between wealth and death risk). The VLW model quantifies both market losses (e.g., income loss) and non-market losses (e.g., value of health status, leisure time) caused by specific diseases through the integration of these two metrics (16, 17). For instance, in 2019, the global VLW due to stroke reached $2,059.67 billion, accounting for 1.66% of global Gross Domestic Product (GDP), with significant regional variations (18) (e.g., 3.01% in Central Europe, Eastern Europe, and Central Asia). Therefore, this study aims to estimate the macroeconomic losses due to breast cancer globally, across 21 super-regions, and in individual countries for the year 2021 using GBD study DALY data.

## 2 Methods

### 2.1 Data sources

The data for this study were derived from GBD database (https://www.healthdata.org/research-analysis/gbd). The GBD database represents a cutting-edge interdisciplinary field integrating public health and data science, aiming to quantify core drivers of human health loss and their spatiotemporal evolution through systematic integration, analysis, and visualization of global health big data. The GBD 2021 study employed machine-learning algorithms to clean heterogeneous data (e.g., incomplete cause-of-death registrations in low-income countries) and used Bayesian statistical models to impute missing values. A spatiotemporal hierarchical regression model was developed to forecast disease-burden trends, and dynamic visualization tools (such as interactive maps and heatmaps) were utilized to intuitively display the complex relationships among the Socio-Demographic Index (SDI), environmental exposures, and health outcomes. Estimations were performed using validated standardized methods, including cause-of-death ensemble modeling for mortality rates, DisMod-MR 2.1 for incidence and prevalence, and spatiotemporal Gaussian process regression for covariates, with age-standardization to the GBD standard population. The study adhered to the GATHER reporting guidelines and cited the GBD 2021 methodological paper for complete technical details (19). All disease terms are standardized using International Classification of Diseases (ICD) codes to ensure accuracy and comparability. This study obtained breast cancer DALYs metrics for 204 countries and territories worldwide from GBD 2021. Uncertainty for all metrics was quantified using Bayesian hierarchical models, with 95% uncertainty intervals calculated based on the 2.5th and 97.5th percentiles from 1,000 posterior distribution samplings to enhance the robustness of statistical inferences. Additionally, 2021 global and national data on GDP, per capita GDP, and Purchasing Power Parity (PPP) were sourced from the World Development Indicators database (https://data.worldbank.org/). The baseline value of a statistical life (VSL) in the United States for 2021 was obtained from the U.S. Department of Transportation’s official estimates (https://www.transportation.gov).

The SDI is a composite metric measuring national multidimensional development levels, constructed by standardizing the geometric mean of three components: lag-distributed income per capita, average education years among the population aged 15 and older, and the total fertility rate for females under 25. According to the GBD classification framework, SDI values range from 0 (theoretical minimum) to 1 (theoretical maximum), with development levels categorized into five tiers—high (0.805–1.000), high-middle (0.689–0.805), middle (0.608–0.689), low-middle (0.455–0.608), and low (0–0.455)—where the thresholds are defined by global population distribution quartiles (13).

### 2.2 Statistical methods

This study conducts a spatiotemporal analysis of the economic burden of breast cancer in 2021 (in 2021 US dollars) using data from the GBD 2021 database and the World Development Indicators database. The VLW serves as the core indicator for assessing the economic burden of disease. A standardized measurement framework is constructed by integrating the VSL and DALYs. VSL is defined as the maximum amount an individual is willing to pay to reduce a specific mortality risk, reflecting the marginal rate of substitution between health preferences and wealth risk. To calculate VLW, VSL data across countries must be standardized, typically using the United States VSL as a benchmark. This involves conversion based on per capita GDP (adjusted for purchasing power parity) and income elasticity (IE), with the introduction of the formula:


$$VSL_{peak,i}=VSL_{peak,USA}{\frac{GDP_{i}}{GDP_{USA}}}^{IE}$$

VSL*_peak,i_* represents the Peak Value of Statistical Life for the *i*-th country or region, defined as the amount people are willing to pay to avoid mortality risks in specific contexts, commonly used to assess life valuation in economic decision-making.


VLWi,t=∑a,sDALYi,a,s t xVPDi,t

where i indexes country, t year, a age group, and s sex; VPD is the value per DALY (USD per DALY). The IE in the formula calibrates cross-country willingness-to-pay variations, typically ranging between 0.55 and 1.5. High-income country conversions (e.g., U.S. to EU) adopt IE = 0.55 as the gold standard, supported by meta-analyses showing stable VSL-income sensitivity at 0.55± in affluent economies (20), while conversions to low-income countries use conservative IE = 1.0 or 1.5 to prevent overestimation of life values from applying low elasticity directly and to reflect reduced health-risk willingness-to-pay due to higher survival consumption shares in low-income populations (WHO recommends IE = 1.0 for high-income and 1.5 for low-income countries) (16, 21). This study employs IE = 1.0 to minimize assumptions about per capita GDP and purchasing-power-adjusted willingness-to-pay, with supplementary analyses using IE = 0.55 and 1.5 enabling readers to apply income-adjusted local willingness-to-pay assumptions. The results of the supplementary analysis with IE = 0.55 and 1.5 are shown in the attachment. We project DALYs to 2050 using the epidemiological model, but hold . nd the income elasticity IE fixed) to express future welfare loss in constant 2021 USD. This isolates the epidemiologic trajectory (DALY) from macroeconomic/valuation growth. In terms of economic evaluation scope, we monetize health loss using VLW = DALYs × VSL (a social welfare indicator). We do not calculate direct medical costs (such as inpatient/outpatient care, medication expenses), non-medical direct costs (transportation, caregiving), or productivity losses. VLW and the components of disease-burden cost (COI) should not be summed; they should be regarded as an independent welfare measure rather than a total budgetary expenditure. All statistical analyses and data visualizations were performed using R (version 4.4.2) and JD_GBDR (V2.37, Jingding Medical Technology Co., Ltd.). In this study, the R software package (version4.2.3) and JD_GBDR (V2.22, Jingding Medical Technology Co., Ltd.) was used for the drawing of the figures.

## 3 Results

### 3.1 Global and 21 super-regions’ economic burden of breast cancer

**Figure 1:**
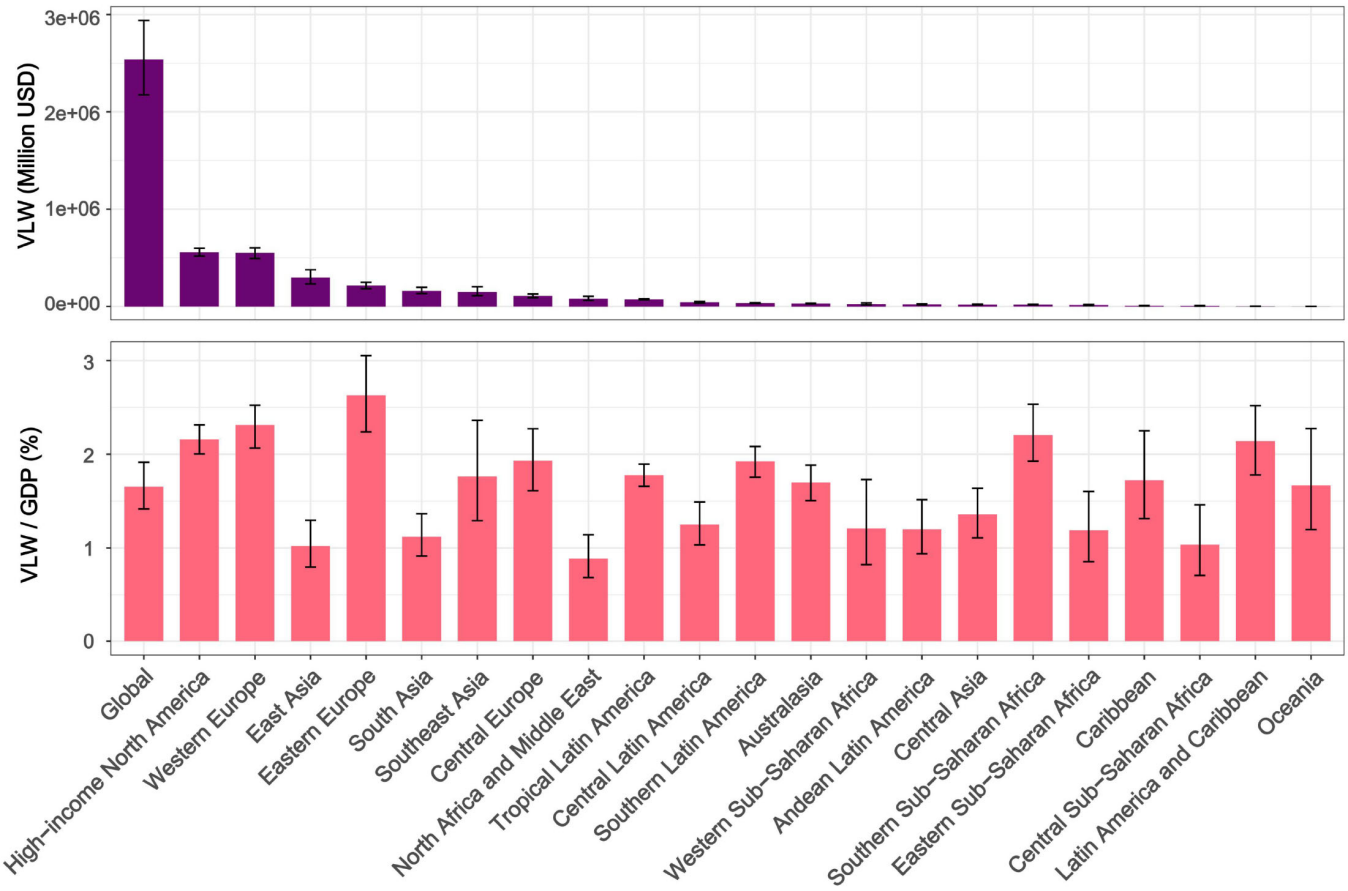
VLW and VLW/GDP of global and regional breast cancer in 2021. VLW, Value of Lost Welfare; GDP, Gross Domestic Product; GBD, Global Burden of Disease.

In 2021, the global total VLW due to breast cancer was 2,538.85 billion USD, accounting for 1.65% of global GDP. The regions with the largest economic losses attributed to breast cancer were High-income North America (557.92 billion USD), followed by Western Europe (551.35 billion USD), East Asia (297.12 billion USD), Eastern Europe (214.788 billion USD), South Asia (161.59 billion USD), Southeast Asia (151.14 billion USD), and others. The three regions with the lowest global breast cancer-related VLW are Oceania (0.96 billion USD), Latin America and Caribbean (2.89 billion USD), and Central Sub-Saharan Africa (6.73 billion USD). Breast cancer-induced VLW accounts for the highest overall share of GDP in Eastern Europe (VLW/GDP = 2.63%) and Western Europe (VLW/GDP = 2.31%). Other regions show Southern Sub-Saharan Africa with an overall breast cancer VLW/GDP of 2.21% (VLW = 19.02 billion USD); High-income North America at 2.16%; Latin America and Caribbean at 2.14% (VLW = 2.89 billion USD); Central Europe at 1.93% (VLW = 108.56 billion USD); and Southern Latin America at 1.92% (VLW = $35.46 billion USD) (**Figure 1**, **Table 1**). In addition, **Tables 2** and **3** respectively present the VLW and VLW-GDP data for the world and for each region in 2021 when IE = 0.55 or 1.5.

**Table 1:** VLW and VLW/GDP of global and regions breast cancer in 2021, IE at 1.0.

Region	VLW ($ billion)	VLW/GDP (%)
Global	2538.85	1.65
Andean Latin America	21.47	1.20
Australasia	29.69	1.70
Caribbean	7.41	1.72
Central Asia	19.46	1.36
Central Europe	108.56	1.93
Central Latin America	42.70	1.25
Central Sub-Saharan Africa	6.73	1.04
East Asia	297.12	1.02
Eastern Europe	214.79	2.63
Eastern Sub-Saharan Africa	14.78	1.19
High-income North America	557.92	2.16
Latin America and Caribbean	2.89	2.14
North Africa and Middle East	81.39	0.89
Oceania	0.96	1.67
South Asia	161.59	1.12
Southeast Asia	151.14	1.76
Southern Latin America	35.46	1.92
Southern Sub-Saharan Africa	19.02	2.21
Tropical Latin America	73.73	1.78
Western Europe	551.35	2.31
Western Sub-Saharan Africa	25.11	1.21

VLW, Value of Lost Welfare; GDP, Gross Domestic Product.

**Table 2:** VLW and VLW/GDP of global and regions breast cancer in 2021, IE at 0.55.

Region	VLW ($ billion)	VLW/GDP (%)
Global	3809.17	2.48
Andean Latin America	42.58	2.38
Australasia	33.10	1.89
Caribbean	16.53	3.85
Central Asia	35.64	2.49
Central Europe	144.49	2.57
Central Latin America	76.41	2.24
Central Sub-Saharan Africa	23.61	3.63
East Asia	525.16	1.8
Eastern Europe	302.11	3.7
Eastern Sub-Saharan Africa	59.12	4.75
High-income North America	563.90	2.18
Latin America and Caribbean	3.75	2.78
North Africa and Middle East	142.91	1.56
Oceania	3.11	5.41
South Asia	445.48	3.09
Southeast Asia	318.36	3.71
Southern Latin America	54.99	2.98
Southern Sub-Saharan Africa	40.42	4.69
Tropical Latin America	141.17	3.4
Western Europe	616.10	2.58
Western Sub-Saharan Africa	82.20	3.96

VLW, Value of Lost Welfare; GDP, Gross Domestic Product.

**Table 3:** VLW and VLW/GDP of global and regions breast cancer in 2021, IE at 1.5.

Region	VLW ($ billion)	VLW/GDP (%)
Global	1854.07	1.21
Andean Latin America	10.1	0.56
Australasia	26.33	1.51
Caribbean	3.56	0.83
Central Asia	10.59	0.74
Central Europe	79.4	1.41
Central Latin America	22.65	0.66
Central Sub-Saharan Africa	1.95	0.3
East Asia	158.25	0.54
Eastern Europe	149.42	1.83
Eastern Sub-Saharan Africa	3.46	0.28
High-income North America	551.81	2.14
Latin America and Caribbean	2.17	1.6
North Africa and Middle East	49.97	0.55
Oceania	0.27	0.48
South Asia	52.83	0.37
Southeast Asia	69.09	0.81
Southern Latin America	21.79	1.18
Southern Sub-Saharan Africa	8.28	0.96
Tropical Latin America	36.28	0.87
Western Europe	492.04	2.06
Western Sub-Saharan Africa	6.82	0.33

VLW, Value of Lost Welfare; GDP, Gross Domestic Product.

### 3.2 The economic burden of breast cancer in different countries

**Figure 2:**
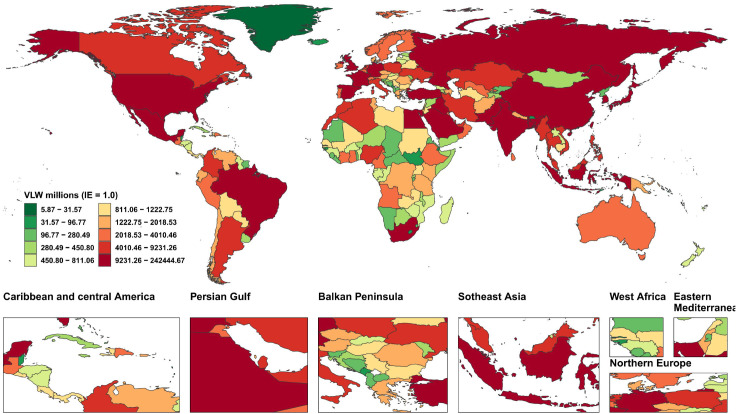
World heat maps of VLW by country for breast cancer in 2021. VLW, Value of Lost Welfare; IE, income elasticity.

**Figure 3:**
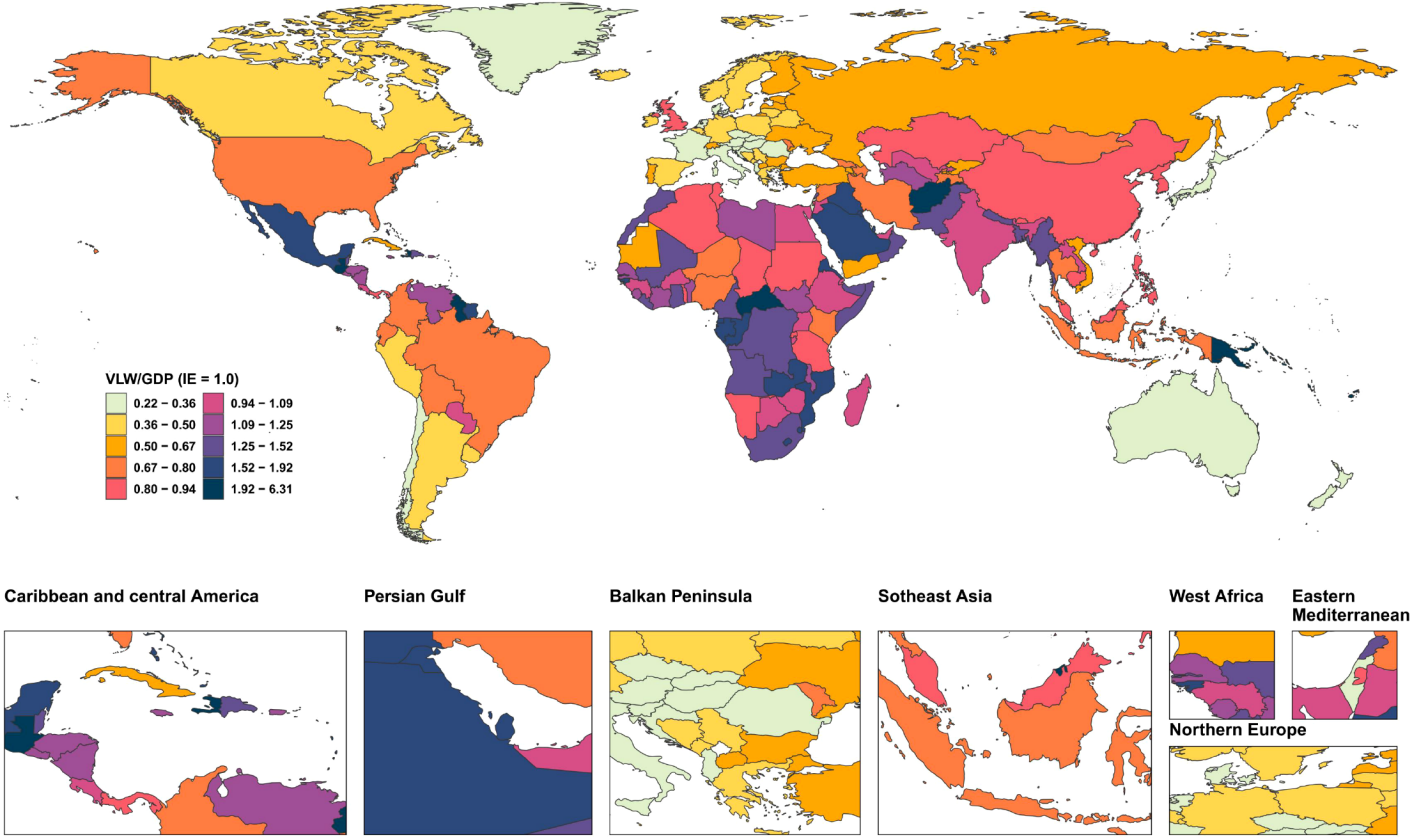
World heat maps of VLW/GDP by country for breast cancer in 2021. VLW, Value of Lost Welfare; GDP, Gross Domestic Product; IE, income elasticity.

We conducted an economic burden analysis of breast cancer across countries worldwide (**Supplementary Tables 1**–**3**). The results show that among all countries, the United States of America faces the highest economic cost of cancer with a VLW value of $518.1 billion USD, followed by China (296.49 billion USD) and the Russian Federation (145.49 billion USD). Other regions with comparatively high costs include India (123.48 billion USD), France (85.25 billion USD), Japan (84.35 billion USD), the United Kingdom of Great Britain and Northern Ireland (78 billion USD), Italy (76.14 billion USD), Brazil (70.26 billion USD), and Indonesia (61.94 billion USD) (**Figure 2**). Regarding the share of VLW attributable to breast cancer as a percentage of GDP, the Principality of Monaco bears the highest economic cost at 5.24% of GDP, followed by Barbados (4.17%), the Republic of Palau (3.92%), the Commonwealth of the Bahamas (3.85%), Georgia (3.68%), Bulgaria (3.57%), American Samoa (3.37%), Serbia (3.34%), Uruguay (3.26%), and Fiji (3.12%). Additionally, the economic cost of breast cancer represents 2.18% of GDP in the United States of America, 1.02% of GDP in China, and 2.58% of GDP in Russia (**Figure 3**).

### 3.3 Breast cancer economic burden across different SDI regions

**Figure 4:**
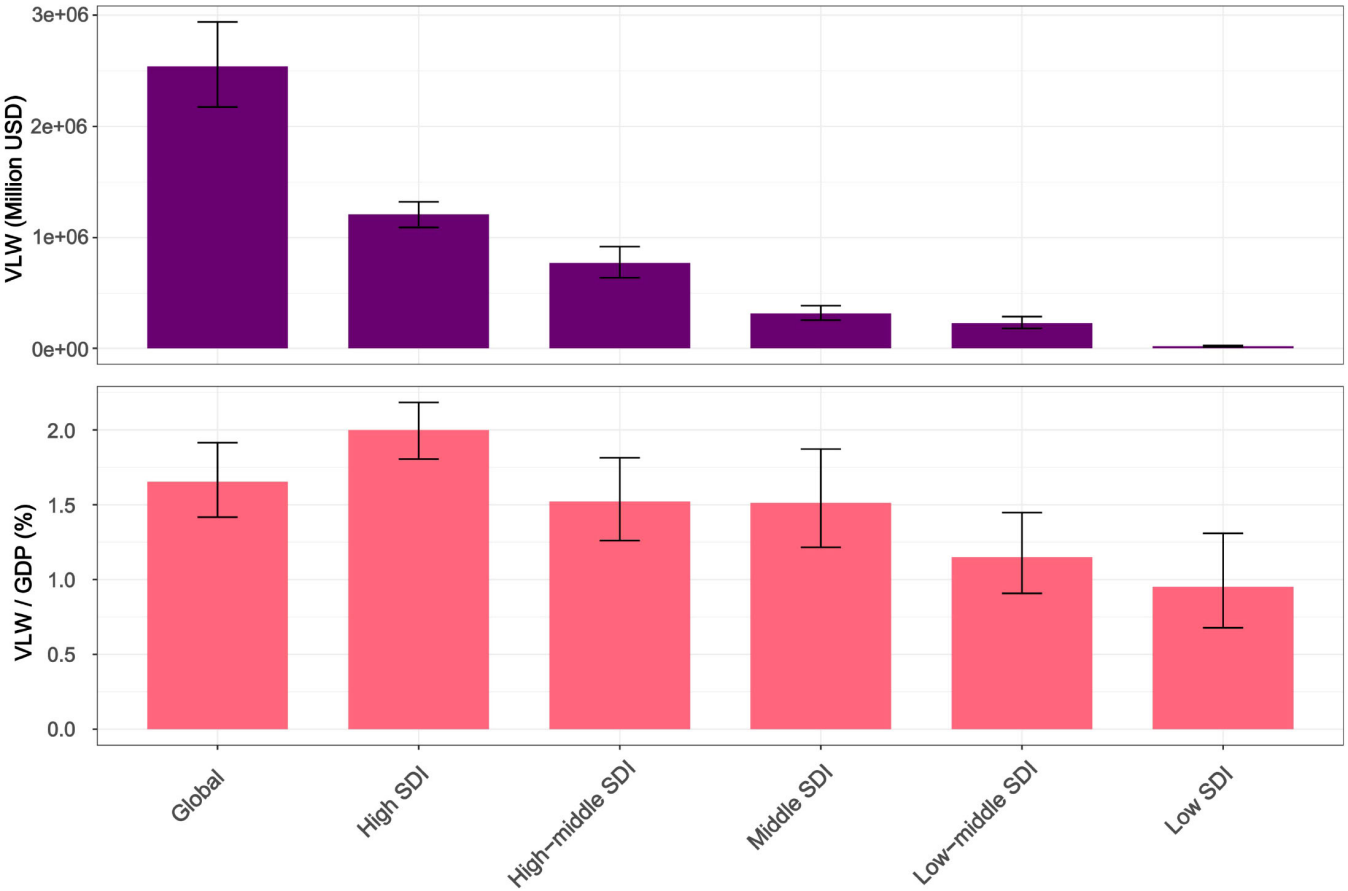
VLW and VLW/GDP of different SDI regions for breast cancer in 2021. VLW, Value of Lost Welfare; GDP, Gross Domestic Product; SDI, Socio-demographic Index.

SDI is a comprehensive indicator used to assess regional development levels, and the breast cancer disease burden varies across different SDI regions (**Figure 4**, **Supplementary Table 4**). Results show that high SDI regions (such as Western Europe, Central Europe, high-income North America, and Australasia) face the highest economic cost of cancer, with VLW overall at 1209.27 billion USD, and the economic cost accounting for 2% of GDP. The economic losses for high-middle SDI, middle SDI, low-middle SDI, and low SDI regions are 771.73 billion US dollars (VLW/GDP =1.52%), 313.88 billion US dollars (VLW/GDP =1.51%), 225.94 billion US dollars (VLW/GDP =1.15%), and 18.04 billion US dollars (VLW/GDP =0.95%) respectively.

### 3.4 Analysis of differences in economic burden of breast cancer by gender and age

**Figure 5:**
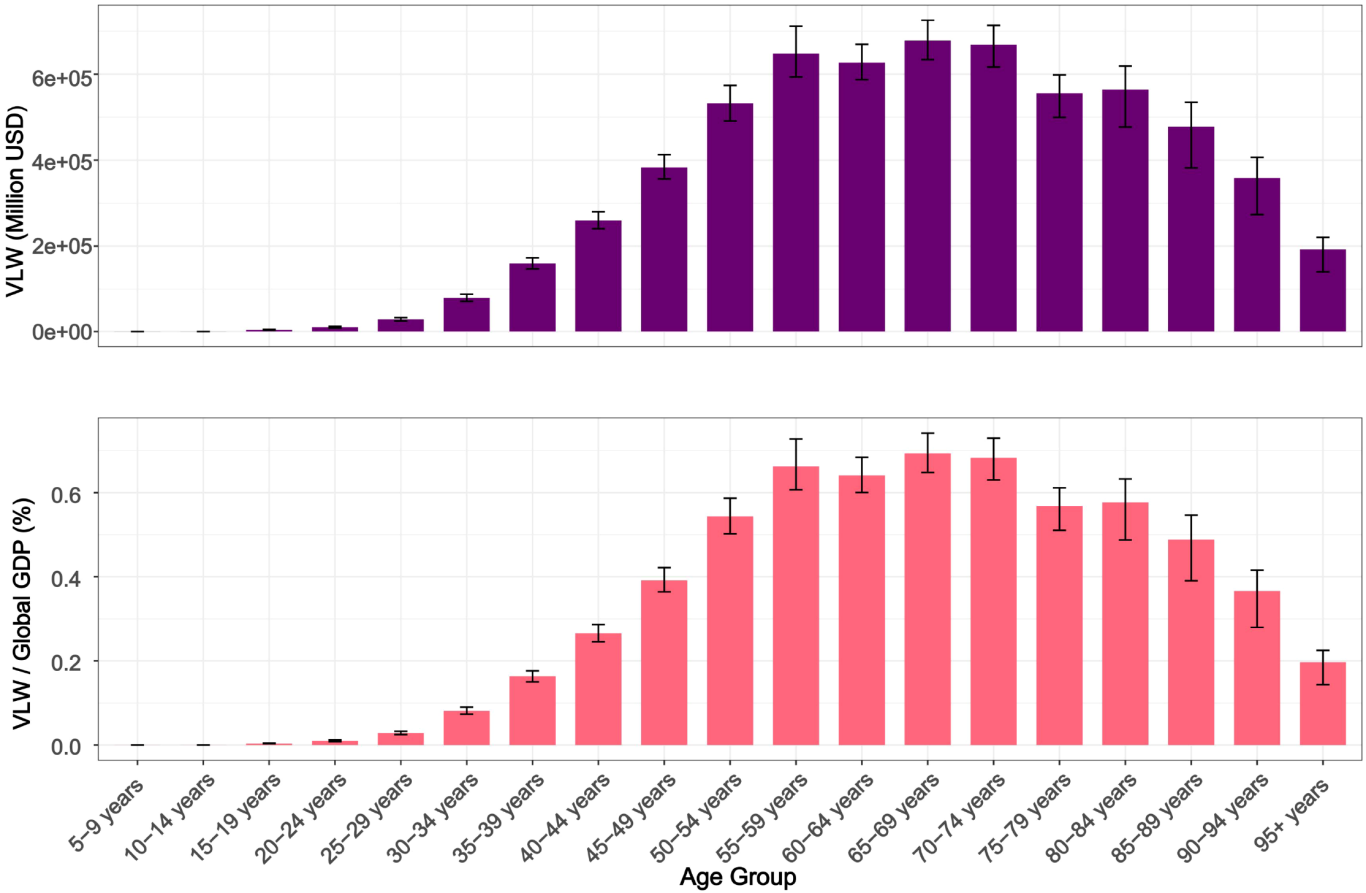
VLW and VLW/GDP of different age groups for breast cancer in 2021. VLW, Value of Lost Welfare; GDP, Gross Domestic Product.

This research team further analyzed disparities in economic losses across different ages and genders(**Figure 5**, **Supplementary Table 5**). The results revealed that as age increases, both the incidence rate and the economic loss caused by breast cancer rise progressively. Notably, the VLW showed a significant surge of 2.83-fold from 28.13 billion USD in the 25-29 age group to 79.63 billion USD in the 30-34 age group. After age 30, the economic loss escalated annually, peaking at 678.25 billion USD (VLW/GDP = 0.69%) in the 65-69 age group before declining gradually. The economic loss for those aged 95 and above was 192.72 billion USD (VLW/GDP = 0.20%). Other age groups—55-59, 60-65, and70-74—also incurred losses exceeding 600 billion USD, specifically 648.08 billion USD (VLW/GDP = 0.66%), 627.17 billion USD (VLW/GDP = 0.64%), and 668.27 billion USD (VLW/GDP = 0.68%), respectively. Regarding gender differences, the total economic loss for females was 2,452.32 billion USD (VLW/GDP = 3.20%), with the peak loss occurring in the 65-69 age group at $612.571 billion (VLW/GDP = 0.63%). For males, the total loss was 37.71 billion USD (VLW/GDP = 0.05%), peaking in the 70-74 age group at 21.23 billion USD (VLW/GDP = 0.02%).

### 3.5 Projected economic burden of breast cancer in 2050

**Figure 6:**
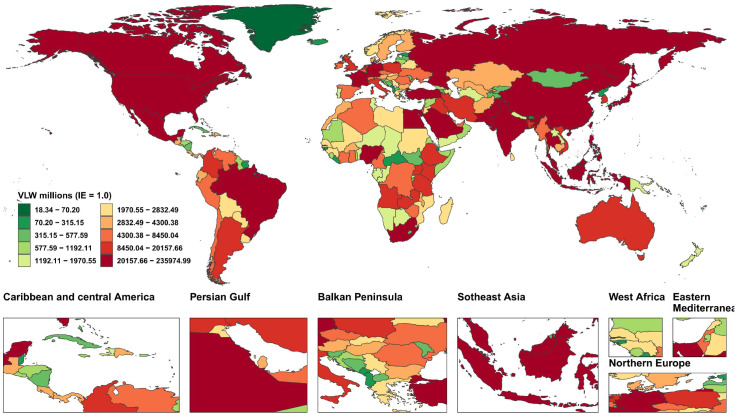
World heat maps of VLW forecast by country for breast cancer in 2050. VLW, Value of Lost Welfare; IE, income elasticity.

**Figure 7:**
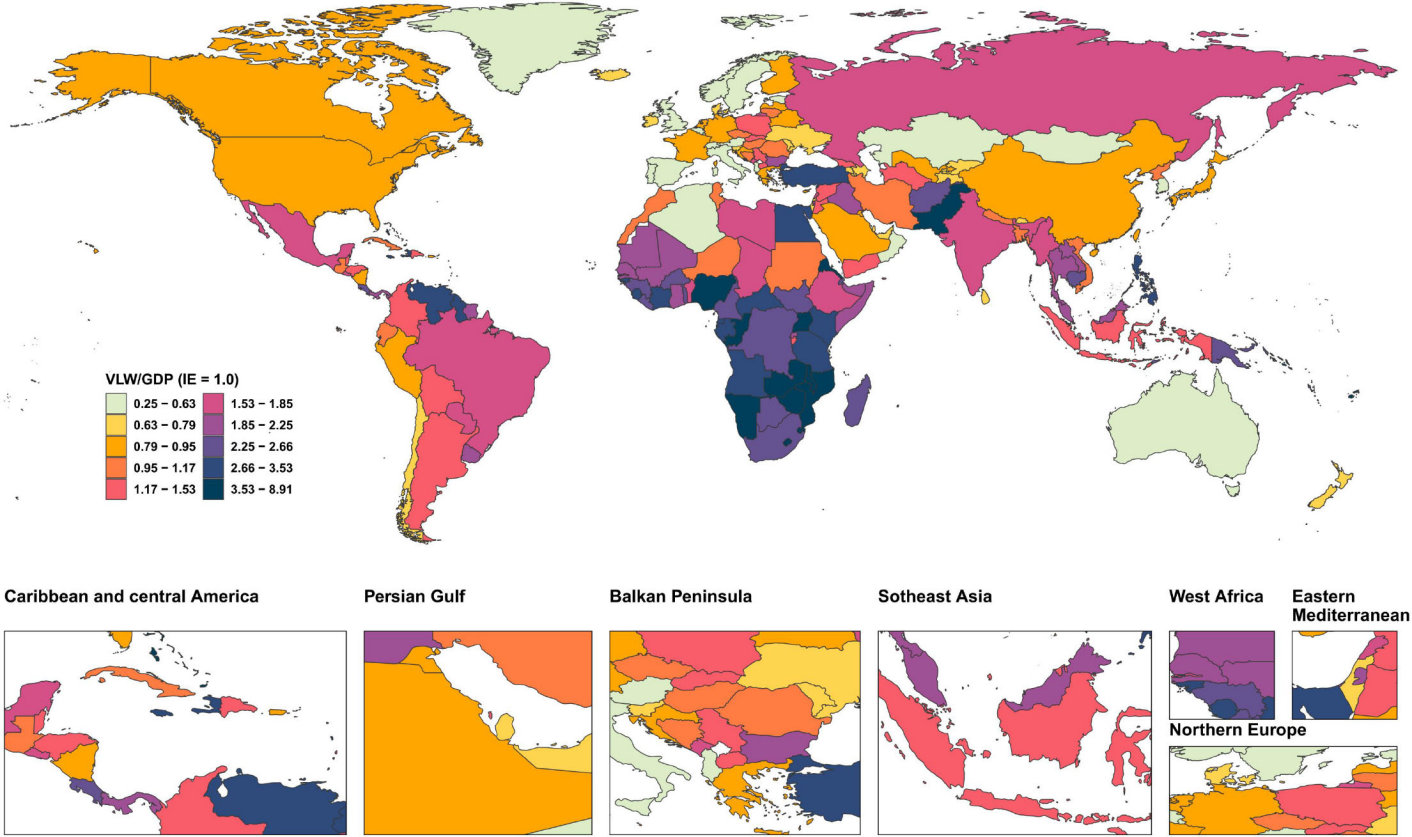
World heat maps of VLW/GDP forecast by country for breast cancer in 2050. VLW, Value of Lost Welfare; GDP, Gross Domestic Product; IE, income elasticity.

We forecasted the economic loss from breast cancer globally, regionally, and nationally for 2050 (**Supplementary Tables 6**–**9**). The results show that global breast cancer-related VLW will reach 2,100.82 billion USD, accounting for 1.28% of global GDP. The region bearing the highest economic loss is South Asia (323.4 billion USD), followed by East Asia (230.64 billion USD), and Southeast Asia (174.69 billion USD). Among all countries, China faces the highest cancer economic cost at 233.64 billion USD, followed by India (210.3 billion USD). Other high-burden countries include Nigeria (95.21 billion USD), the Russian Federation (79.72 billion USD), Brazil (67.24 billion USD), and Indonesia (54.92 billion USD) (**Figure 6**). Regarding breast cancer-related VLW as a percentage of GDP, Zimbabwe has the highest share at 8.82%, followed by Lesotho (7.51%), Malawi (7.03%), Zambia (6.78%), American Samoa (6.7%), Bulgaria (3.57%), American Samoa (3.37%), Serbia (3.34%), Uruguay (3.26%), and Fiji (3.12%). Additionally, the economic cost as a percentage of GDP is 0.81% for the United States of America, 0.90% for China, and 1.72% for India (**Figure 7**).

## 4 Discussion

The GBD database systematically elucidates the global epidemiological evolution of breast cancer, revealing the multidimensional heterogeneity in its incidence, mortality, and risk characteristics. As the most frequently diagnosed malignancy among women globally, breast cancer accounts for 25% of female cancers and causes 689,000 deaths annually (representing 6.9% of total cancer deaths) (19, 22). Between 1990 and 2021, the age-standardized incidence rate (ASIR) of breast cancer increased from 39.99 to 46.40 per 100,000 population (19). However, growth rates showed significant regional disparities, with SDI regions like North America reaching an ASIR of 94.93 per 100,000-3.8 times higher than low-SDI regions such as South Asia (24.62 per 100,000). High-income regions like North America and Western Europe consistently exhibited ASIRs above the global average (4, 19). Conversely, the age-standardized mortality rate (ASMR) in high-SDI regions declined from 16.60 per 100,000 in 1990 to 14.55 per 100,000 in 2021 (19). In SDI countries/regions (e.g., North America, Western Europe), the growth rate of disease incidence has slowed due to widespread screening coverage (exceeding 80%) and population aging (23). Conversely, in low-to-middle SDI countries/regions (e.g., sub-Saharan Africa), the ASIR has increased annually by 3%–5% due to Westernized lifestyles and inadequate healthcare resources, with countries like Malawi experiencing increases exceeding 5% (24). Our team’s preliminary research identified the main mortality drivers for breast cancer as smoking, second-hand smoke, high BMI, alcohol consumption, red-meat intake, elevated fasting blood glucose, and lack of physical activity. Among these, a high red-meat diet contributes the highest attributable share of ASMR. In high-SDI regions, the primary contributors are alcohol consumption (population-attributable proportion: 21.3 %) and red-meat intake (19 %–20 %). In low-SDI regions, the major challenges are exposure to second-hand smoke (risk increase >30 %) and the elevated risk associated with alcohol consumption (8). This epidemiological divergence directly leads to a “development paradox”: High-SDI regions account for 75% of global cases but only 40% of deaths, while low-SDI regions, despite having fewer cases, exhibit extremely high mortality rates (low-SDI countries: 16.00/100,000 vs. high-SDI countries: 15.44/100,000) (19). The core driver lies in disparities in early diagnosis and treatment access: the early diagnosis rate exceeds 70% in high-SDI countries, whereas in low-income countries, only 25% have access to cancer surgical services (2, 25). Furthermore, the proportion of young patients is as high as 44.1%, exacerbating labor force losses. The economic burden of breast cancer exhibits three distinct characteristics: a high initial peak at diagnosis, persistent long-term follow-up costs, and significant regional/subtype variations. Consequently, the disease burden and economic impact of breast cancer reveal profound global inequalities and dynamic evolution. International studies indicate that direct medical costs in the first year of diagnosis typically peak (approximately 10,000 annually in Australia beyond 5 years), and about 10% of patients face severe financial hardship due to treatment (26). The longitudinal distribution of economic burden is jointly influenced by disease stage, molecular subtype, healthcare accessibility, and healthcare insurance policies. This study quantified macroeconomic welfare loss using VLW (DALYs × VSL, 2021 Int$) and qualitatively discussed implications for therapy affordability and policy levers.

Breast cancer impacts the global economy through three mechanisms: direct medical costs, productivity losses, and inhibition of capital investment. In 2021, the global VLW reached 2,538.849 billion US dollars, accounting for 1.65% of global GDP, but its regional distribution was highly uneven. High-income North America had the highest VLW (557.919 billion USD), followed by Western Europe (551.354 billion USD), while Africa accounted for only 0.24%. The high economic burden in high-income regions stems from three pressures: first, fundamental differences in medical cost structures, where targeted drugs (such as CDK4/6 inhibitors costing over $100,000 annually) and extensive screening drive up medical costs (27, 28), for example, in the US, the average monthly medical expenditure for metastatic recurrent patients is $8,575 higher than for non-recurrent patients (11); second, population aging intensifies long-term care needs, although DALYs for people over 70 have decreased by 15% through early intervention, care costs continue to rise (29); third, the incidence rate remains high, with the ASIR in SDI countries reaching 77.08 per 100,000, which is 2.7 times that of low-income countries. However, high investment also brings efficiency improvements—the mortality rate in high-SDI countries is only 8.54 per 100,000, significantly lower than in low-income countries. This reflects the effectiveness of their screening coverage exceeding 80% and advanced treatment systems. For example, breast cancer screening coverage in England reaches 85.5%, while in Bangladesh it is only 1.7% (30).

In sharp contrast, there is a hidden economic disaster in low-income regions. In low-SDI regions such as sub-Saharan Africa, the absolute value of breast cancer VLW is only $19.022 billion USD, but the relative impact on families and society is more severe. Firstly, the disease affecting younger populations leads to labor force disruption, with 47% of patients under 50 years old (globally, 71% of cases occur in those over 50), but the return-to-work rate is lower among low-income groups. For example, low-income individuals often have jobs requiring physical labor (such as heavy lifting) or lack flexible arrangements, accompanied by no paid sick leave, absence of benefits, and employers’ perceived discrimination reduces the probability of returning to work by 73% (31) (32). Secondly, the cost structure dominated by late-stage diagnosis and treatment distorts resource allocation. Due to pathological diagnosis coverage being below 10%, patients are often diagnosed at advanced stages (33). Palliative care-related caregiver income loss accounts for 58% of total household expenditures, far exceeding direct medical costs (33). In Africa, the cost of treating breast cancer far exceeds the affordability of average households and society. A study in Nigeria reported that approximately 85% of diagnosed breast cancer patients are low-income, with 70% earning less than ₦12,500.00 (~$100) annually. Yet, every three weeks, patients accumulate bills of ₦150,000–₦350,000 (~$962–1=₦156) (34). Moreover, access to key prevention and treatment tools remains extremely low. In Nigeria and Uganda, 38% of patients abandon treatment (including medications) due to high costs, particularly among low-income groups. While tamoxifen is relatively inexpensive, overall medication expenditures account for a high proportion of household income, with accessibility remaining below 20% (35). The characteristic of “low absolute value, high relative burden” triggers a vicious cycle—healthcare expenditure crowds out investment in education and infrastructure, further undermining economic development potential. The root cause of the increased burden in low-income regions lies in the dual failure of risk exposure and the healthcare system. Metabolic risks have become the primary driver—the attributable risk of high fasting blood glucose has surged by 152%, and metabolic diseases such as high BMI are proliferating, yet basic management of chronic diseases is severely deficient (36); the failure of medical technology adaptation exacerbates the situation, with only approximately 2.2% of women aged 40-69 in low- and middle-income countries (LMICs) having undergone breast cancer screening (including mammography), and countries like Malawi lacking mammography equipment, relying primarily on clinical palpation for screening, but the coverage of clinical palpation is also limited (37). “Deepening the issue are data black holes and policy lags. In North America, cancer registration coverage is as high as 83%-97.2%, in Europe it is 32%-66.4%, but in Africa coverage is only 2%-5.3%, in some parts of Asia less than 10%, and in Central and South America generally below 10% (38). This leads to age-standardized breast cancer incidence rates in Namibia and Nigeria rising to 127 per 100,000 and 71.1 per 100,000, respectively. However, in LMICs, breast cancer screening and early diagnosis services are often not included in national core health plans (36). Moreover, the imbalance in global governance has further widened the disparity, with LMICs bearing 80% of the global cancer burden but receiving only 5% of cancer funding (39); patent barriers prevent 90% of targeted drugs from entering these markets, making even the WHO-recommended cost-effectiveness threshold of $500 per quality-adjusted life year an unattainable dream (40).

Gender and age dimensions also reveal disparities in burden distribution. The age-standardized incidence rate of male breast cancer, although below 1 per 100,000, increased more rapidly between 1990 and 2021. Delayed diagnosis due to insufficient disease awareness exacerbates treatment costs (41). Women, however, bear broader productivity losses, with breast cancer causing significantly higher per capita DALYs loss in women compared to men, particularly in low-income countries—where women simultaneously fulfill dual roles in economic production and family caregiving, leading to a severe collapse in household economic resilience when afflicted by the disease (42). The age distribution indicates that individuals aged ≥50 years account for 71% of global cases. However, in sub-Saharan Africa, 47% of new cases are concentrated in those under 50 years (43). In China, the annual growth rate of incidence among women under 40 years old is 3.7%, significantly higher than that in the elderly group (2%) (44). Additionally, both the ASIR and ASMR show a clear upward trend with increasing age. Age-stratified data reveals that in countries with SDI, the combination of screening with targeted therapies and endocrine therapy has significantly reduced breast cancer DALYs among individuals aged 70 and older. However, in low-income countries, inadequate geriatric healthcare has led to an annual increase of 1.55% in the ASIR for patients over 70 (45). Among women of reproductive age (15–49 years), breast cancer incidence disproportionately affects low-income regions, where deaths result in “orphan care costs.” For instance, in Sub-Saharan Africa, breast cancer mortality generates 121 orphans per 100 deaths, with 85% under 18 years old and 51% aged 10–17, imposing long-term social welfare burdens (46).

By 2050, the global economic burden of breast cancer is projected to undergo structural shifts, with the total amount decreasing to $2,100.82 billion (1.28% of global GDP) while experiencing significant regional redistribution. South Asia will emerge as the highest economic cost region ($323.4 billion), followed closely by East Asia ($234.25 billion), together accounting for 26.5% of the global burden. China ($233.64 billion) and India ($210.3 billion) as the most burdened nations, reflecting the diffusion of population growth, accelerated aging, and “Westernization” of risk factors (e.g., high BMI, low fertility, alcohol consumption) in low- and middle-income countries. Although absolute costs remain lower in Sub-Saharan Africa, mortality rates in Namibia, Nigeria, and others are rising by >5% annually, potentially pushing the VLW/GDP ratio beyond 3%. The proportion of breast cancer burden in North America decreased from 22% in 2021 to 11% in 2050, and in Western Europe from 21.7% to approximately 14%. This decline is attributed to the widespread screening (mammography screening rate in North America >70%) and advances in targeted therapies (30). However, the annual per capita treatment cost for metastatic breast cancer in the United States is as high as $170,000, continuously driving up societal indirect costs (47). Notably, micro-economies (e.g., Monaco, Barbados) may sustain extreme VLW/GDP levels of 4–5% due to limited healthcare resources.

The VLW framework has for the first time enabled globally standardized comparisons of the economic burden of breast cancer, revealing a dual-track dilemma where high absolute burdens concentrate in high-SDI countries while high relative burdens devastate low-SDI households; however, its inherent flaws mirror global health inequities—VSL assumptions disregard payment capacity disparities, and DALY weights embed cultural hegemony. Our analysis does not estimate direct medical expenditures or other COI components due to cross-country data comparability constraints; VLW complements, but does not replace, payer- or provider-perspective cost studies. Future evolution requires advancing the framework through data justice (e.g., community-participatory disability weight formulation) and dynamic VSL models (integrating micro-level willingness-to-pay surveys); otherwise, it risks exacerbating the Matthew effect in resource allocation.

In summary, narrowing the breast cancer burden gap hinges on moving beyond the unidirectional “high investment–high efficacy” mindset. High-SDI countries must guard against cost escalation driven by technological dependency, while low-to-middle-SDI countries should avoid inefficient duplication of resources. Short-term priorities include reducing endocrine drug prices (e.g., tamoxifen) to ≤$10/month via medicine patent pools. Mid-term strategies should replace fixed screening devices with portable ultrasound to enhance grassroots accessibility. Long-term solutions require integrating breast cancer control into maternal health systems—exemplified by Ethiopia’s rural program leveraging existing prenatal care channels to cover 56% of women of reproductive age (48). The core lies in restructuring the cost-effectiveness evaluation framework: CBE combined with health education can achieve a mortality reduction similar to mammography (16.3%), while costing only half as much. This strategy has been proven more feasible in studies from Ghana, India, and other locations (49). Global collaborative mechanisms also require innovation. Examples include imposing cross-border tobacco taxes (a 1% increase could raise $14 billion annually) to fund medicine procurement in Africa, or requiring pharmaceutical companies to contribute 0.1% of breast cancer drug sales to a global fund. Upgrading data systems relies on the Global Initiative for Cancer Registry Development to establish regional hubs (e.g., a Latin American hub) for technical support, training, and promotion of free software (e.g., CanReg5) to standardize data management, thereby improving data quality in low- and middle-income countries (50). Only by transforming economic burden analyses into differentiated investment strategies can we reverse the “late diagnosis → workforce loss → worsening poverty → lack of medical care” death spiral in low-income regions and achieve the WHO’s 2040 goal of saving 2.5 million lives.

## Data Availability

The original contributions presented in the study are included in the article/**Supplementary Material**. Further inquiries can be directed to the corresponding authors.
