# Supplementary material for: Estimates and projections of the global economic cost of breast cancers from 2021 to 2050
Source: Front Endocrinol (Lausanne). 2025 Dec 4;16:1692619. doi: 10.3389/fendo.2025.1692619 (PMC12711471; doi:10.3389/fendo.2025.1692619)
Supplement: Supplementary file 1 [file DataSheet1.doc]

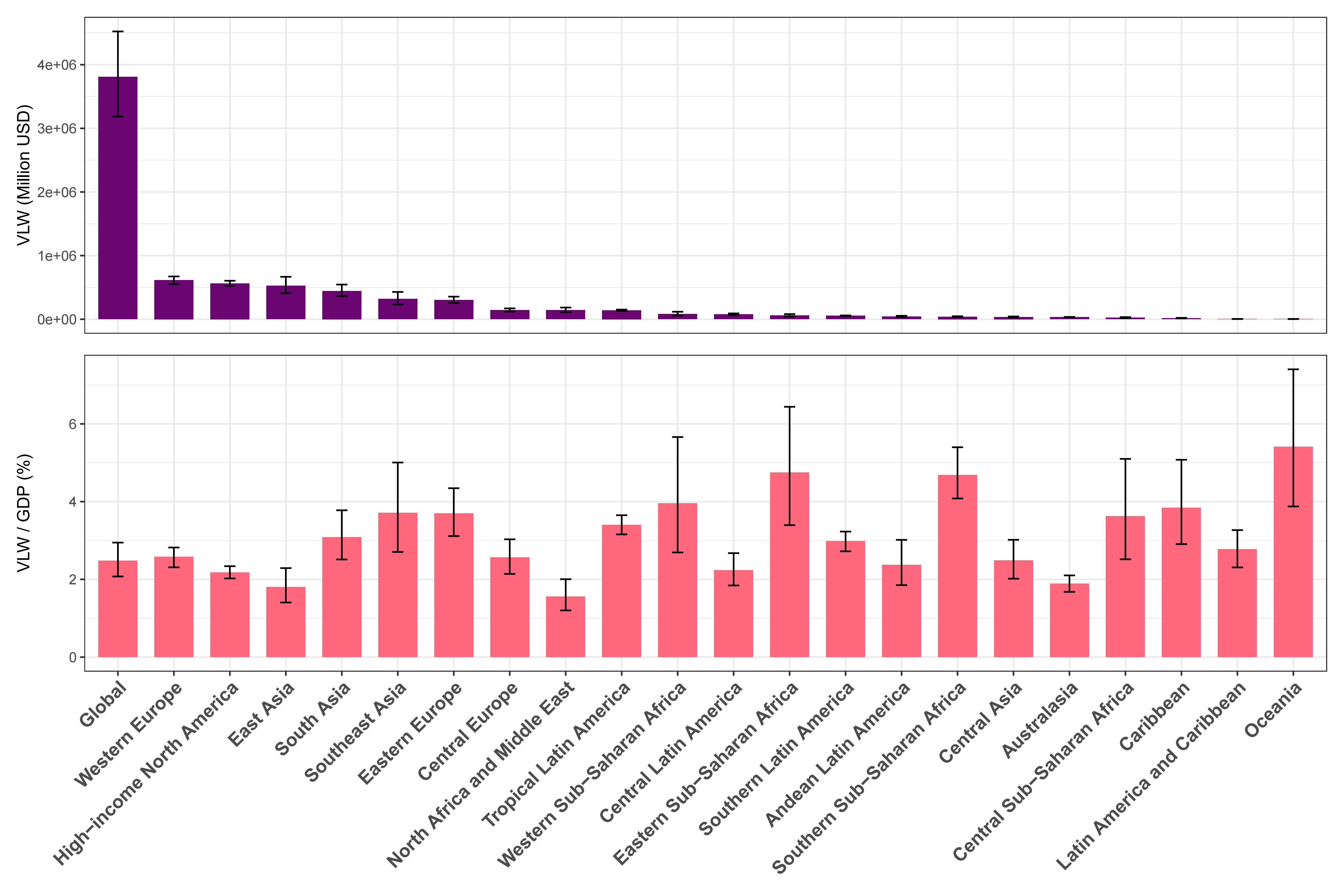


**A**

**B**


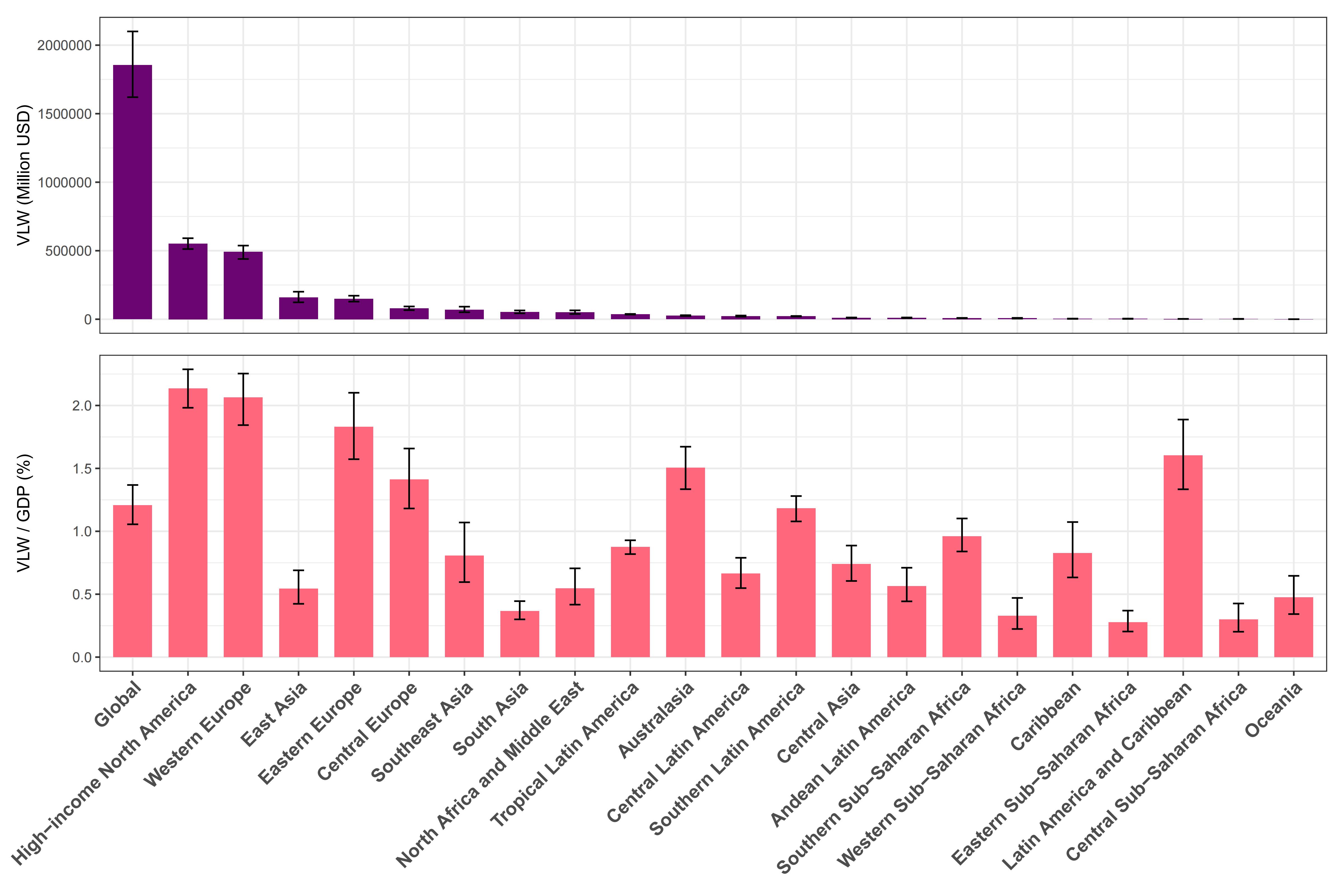


Supplementary Figure 1. VLW and VLW/GDP of global and regional breast cancer in 2021. A using IE at 0.55, B using IE at 1.5; VLW, Value of Lost Welfare; GDP, Gross Domestic Product; GBD,Global Burden of Disease; PPP, Purchasing Power Parity; IE, income elasticity


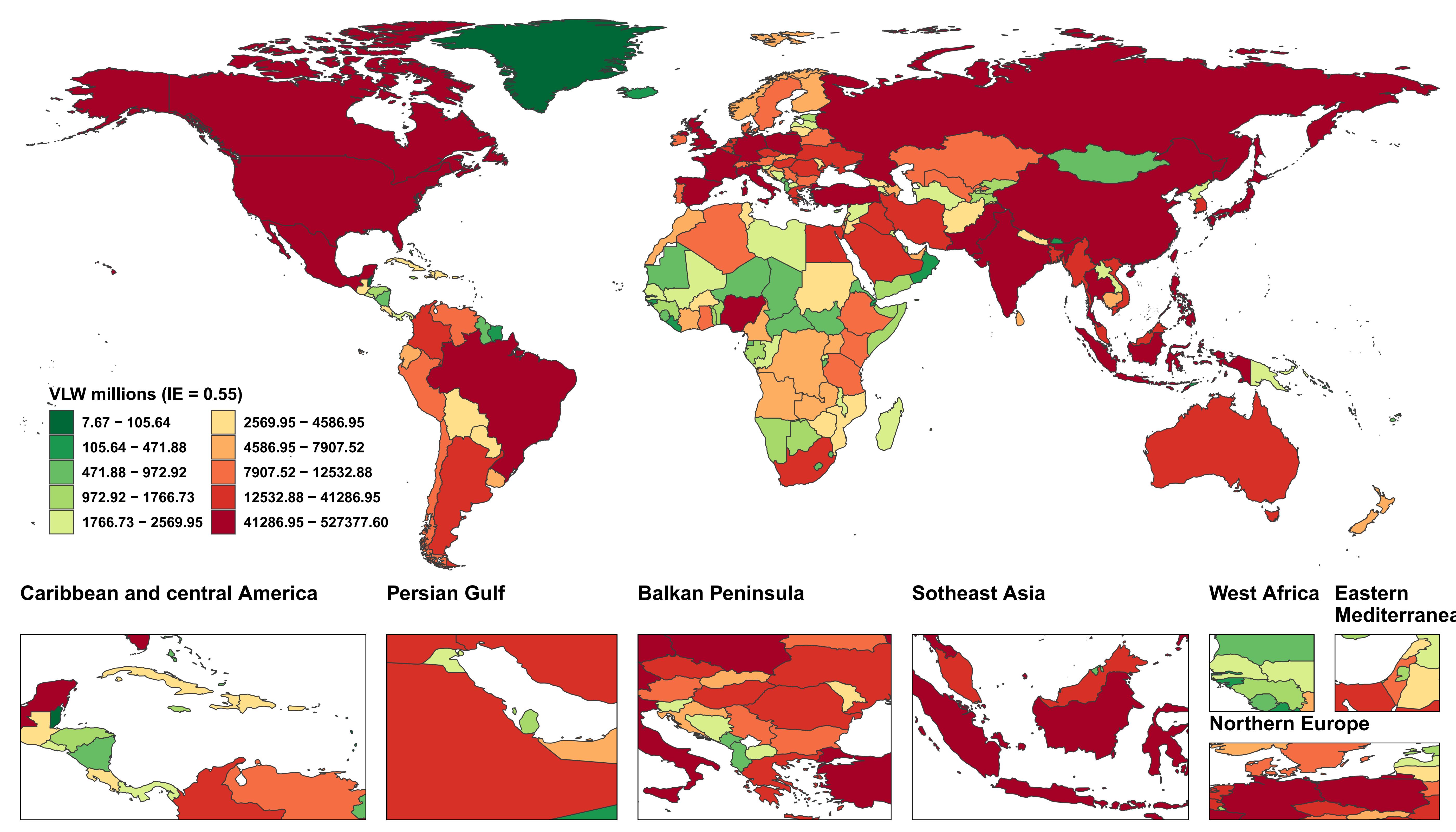

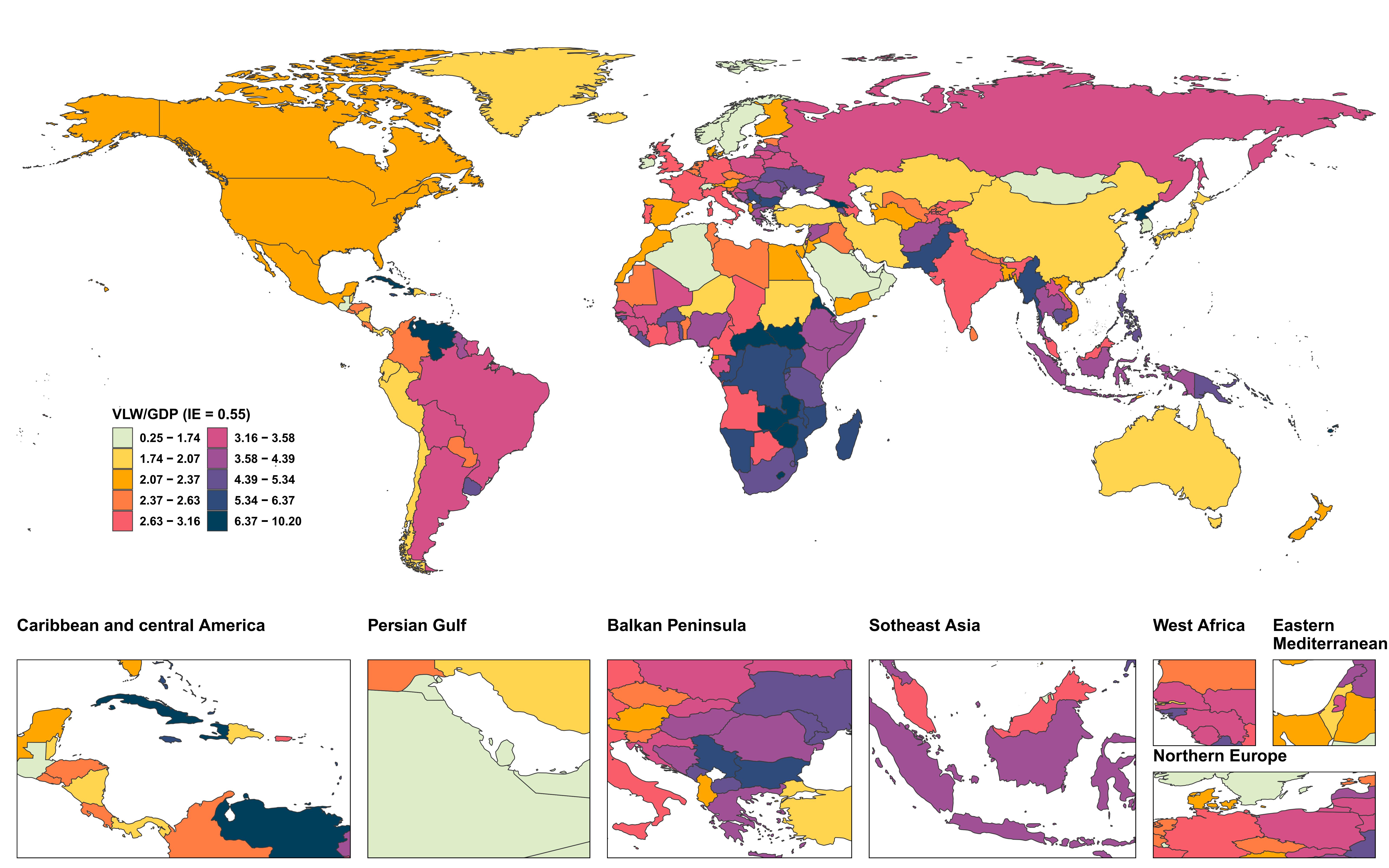


B

A

Supplementary Figure 2. World heat maps of VLW and VLW/GDP by country for breast cancer in 2021, using IE at 0.55. A is the world heat map of VLW; B is the world heat map of VLW. VLW, Value of Lost Welfare; GDP, Gross Domestic Product; GBD,Global Burden of Disease; PPP, Purchasing Power Parity; IE, income elasticity


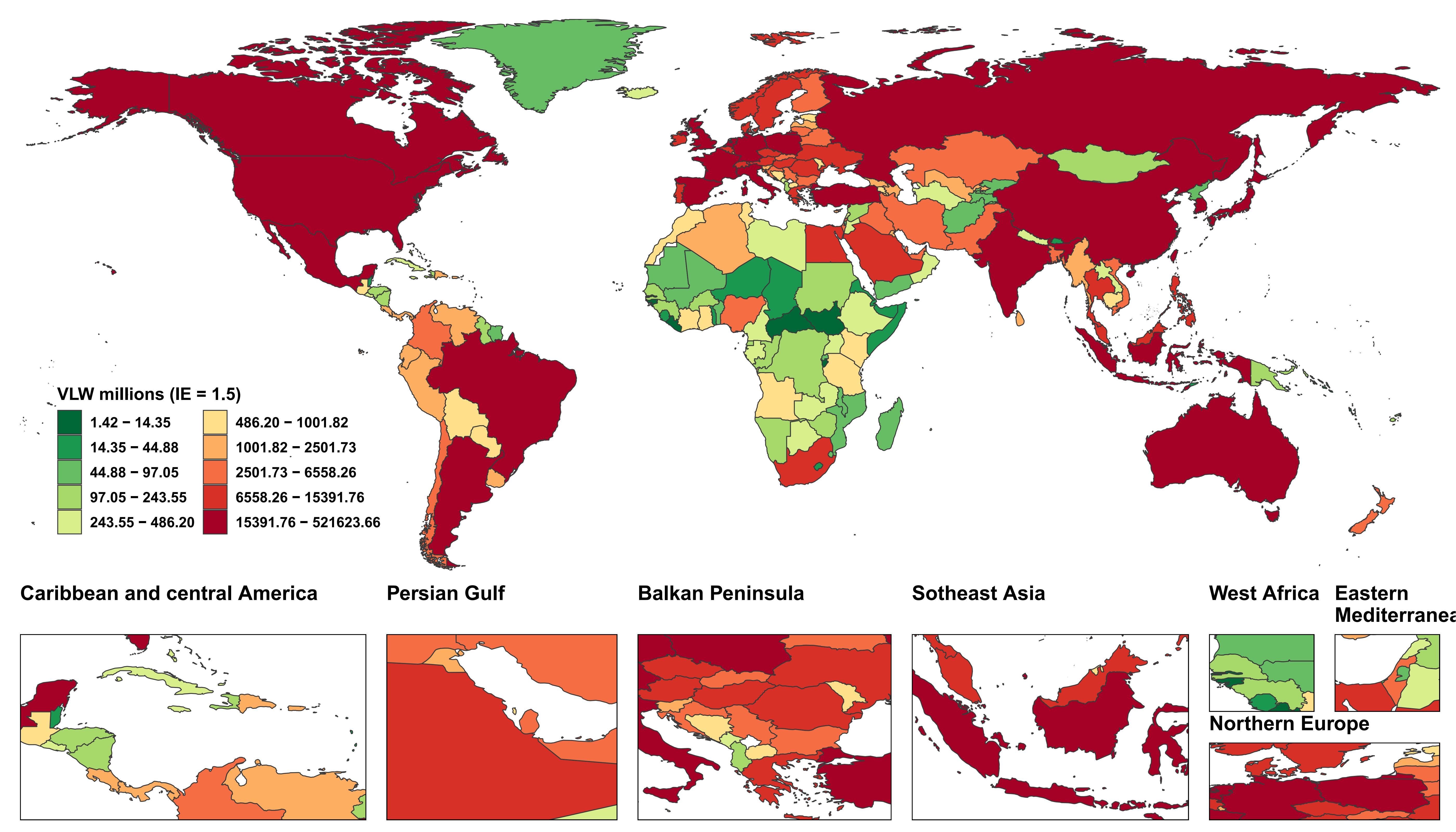

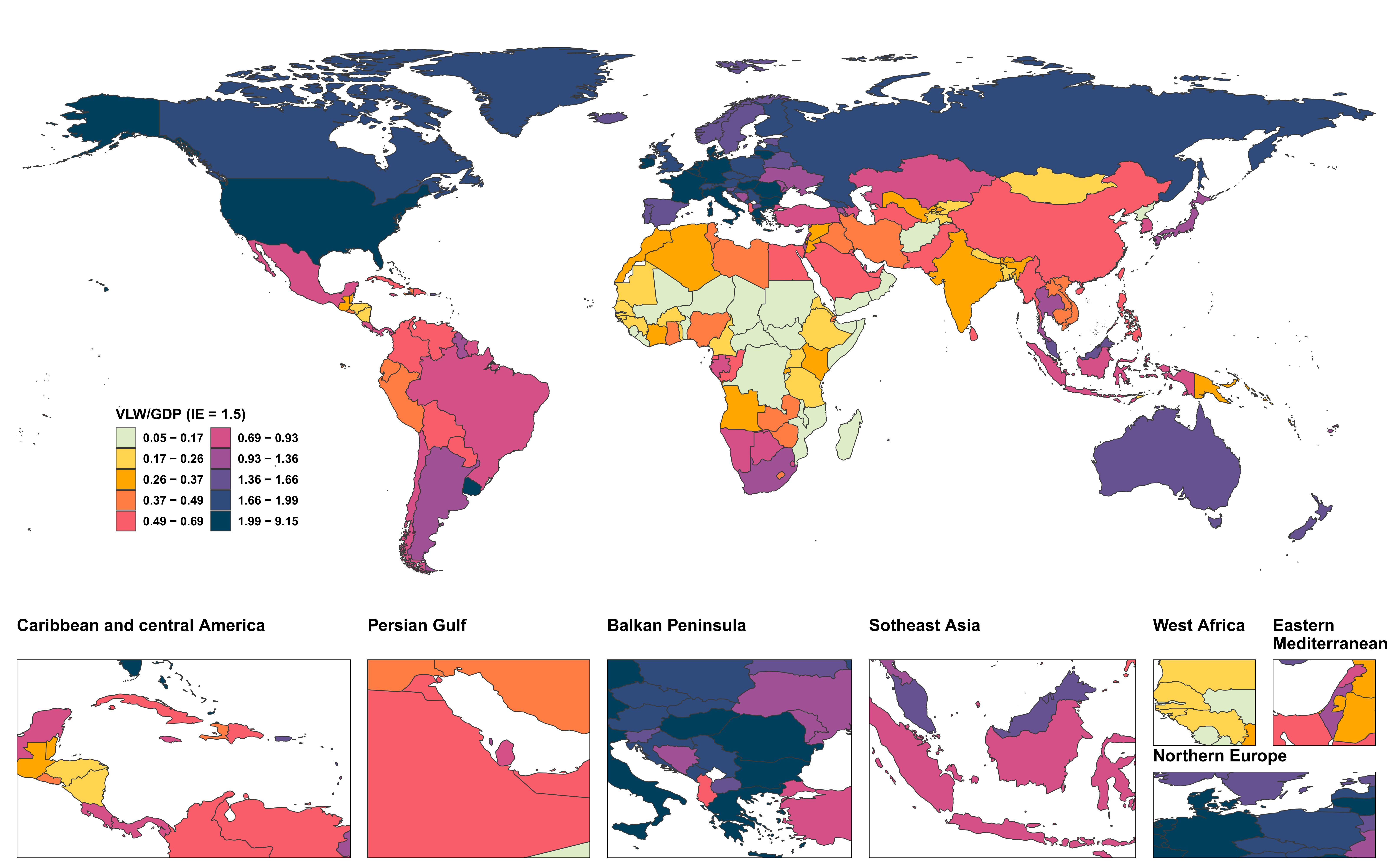


A

B

Supplementary Figure 3. World heat maps of VLW and VLW/GDP by country for breast cancer in 2021, using IE at 1.55. A is the world heat map of VLW; B is the world heat map of VLW. VLW, Value of Lost Welfare; GDP, Gross Domestic Product; GBD,Global Burden of Disease; PPP, Purchasing Power Parity; IE, income elasticity


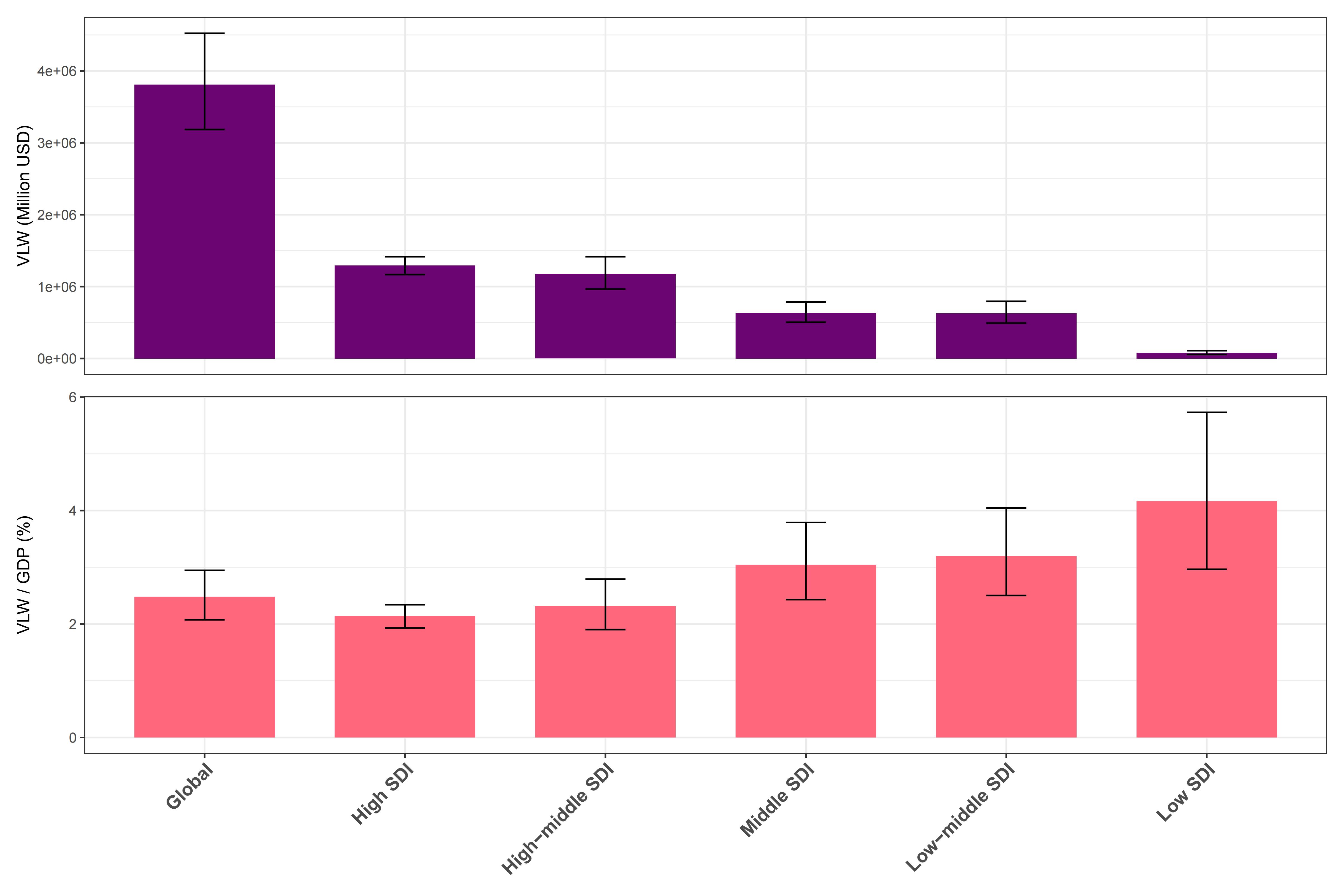

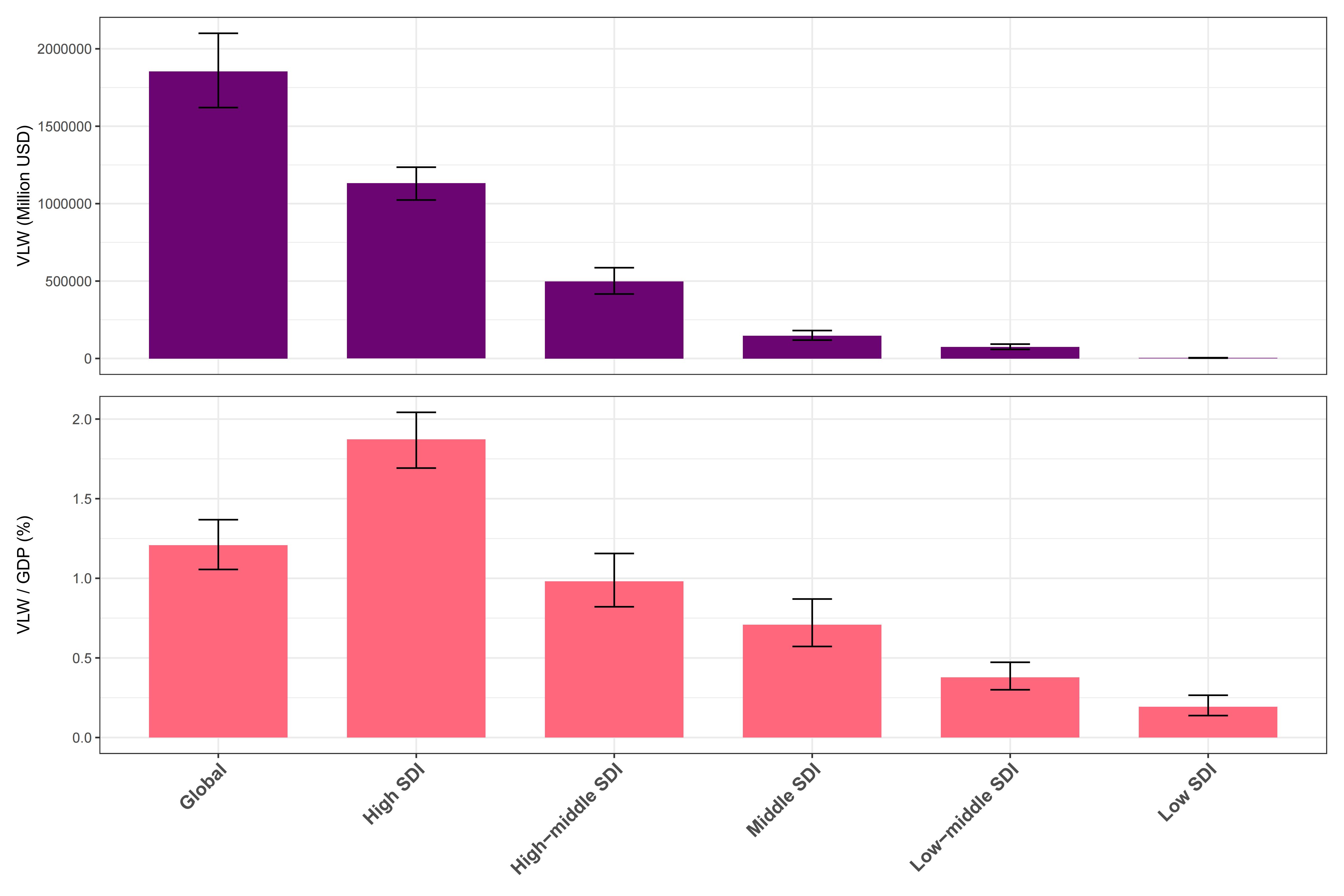


B

A

Supplementary Figure 4. VLW and VLW/GDP of different SDI regionsl for breast cancer in 2021.A using IE at 0.55, B using IE at 1.5; VLW, Value of Lost Welfare; GDP, Gross Domestic Product; GBD,Global Burden of Disease; PPP, Purchasing Power Parity; IE, income elasticity;SDI, Socio-demographic Index


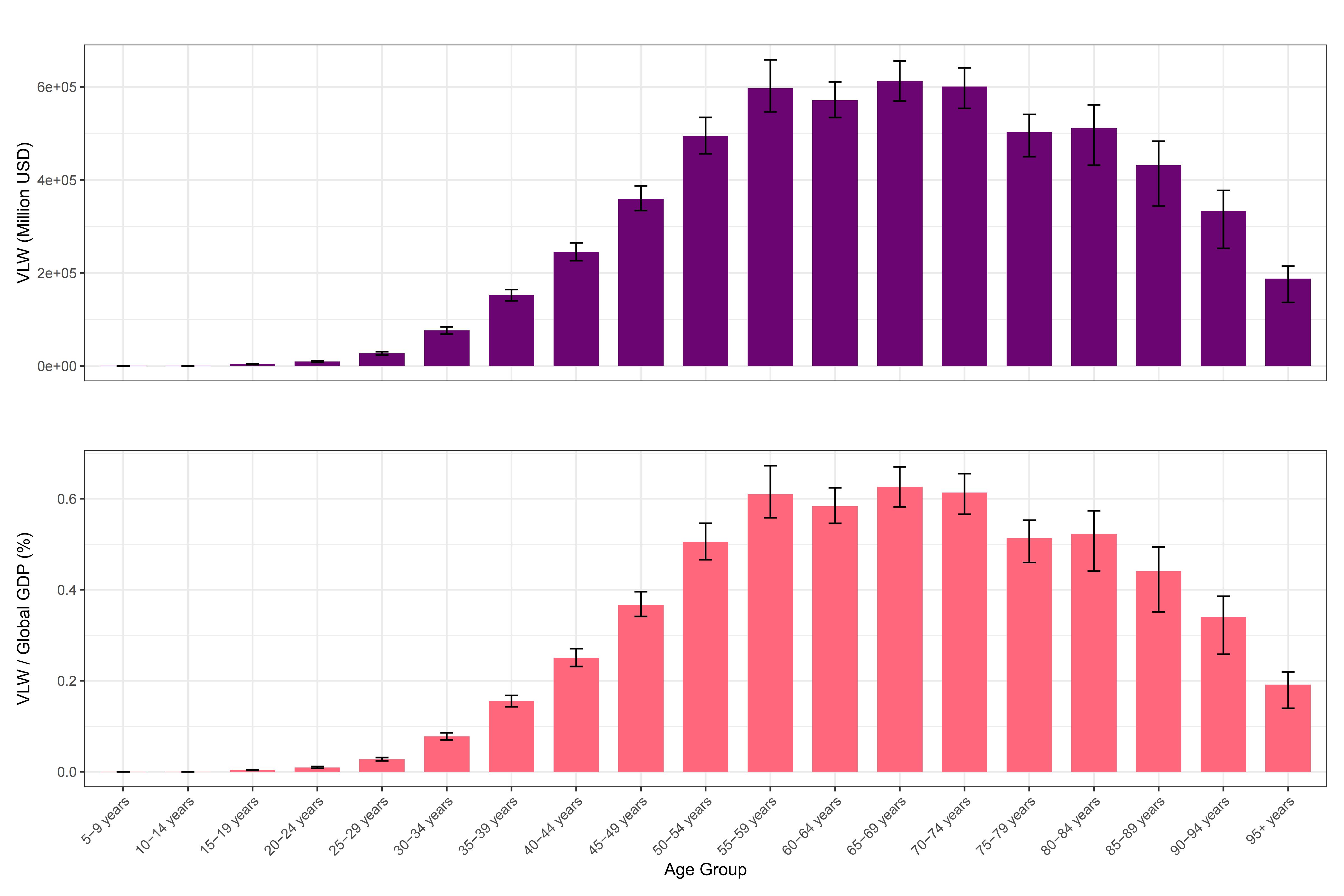

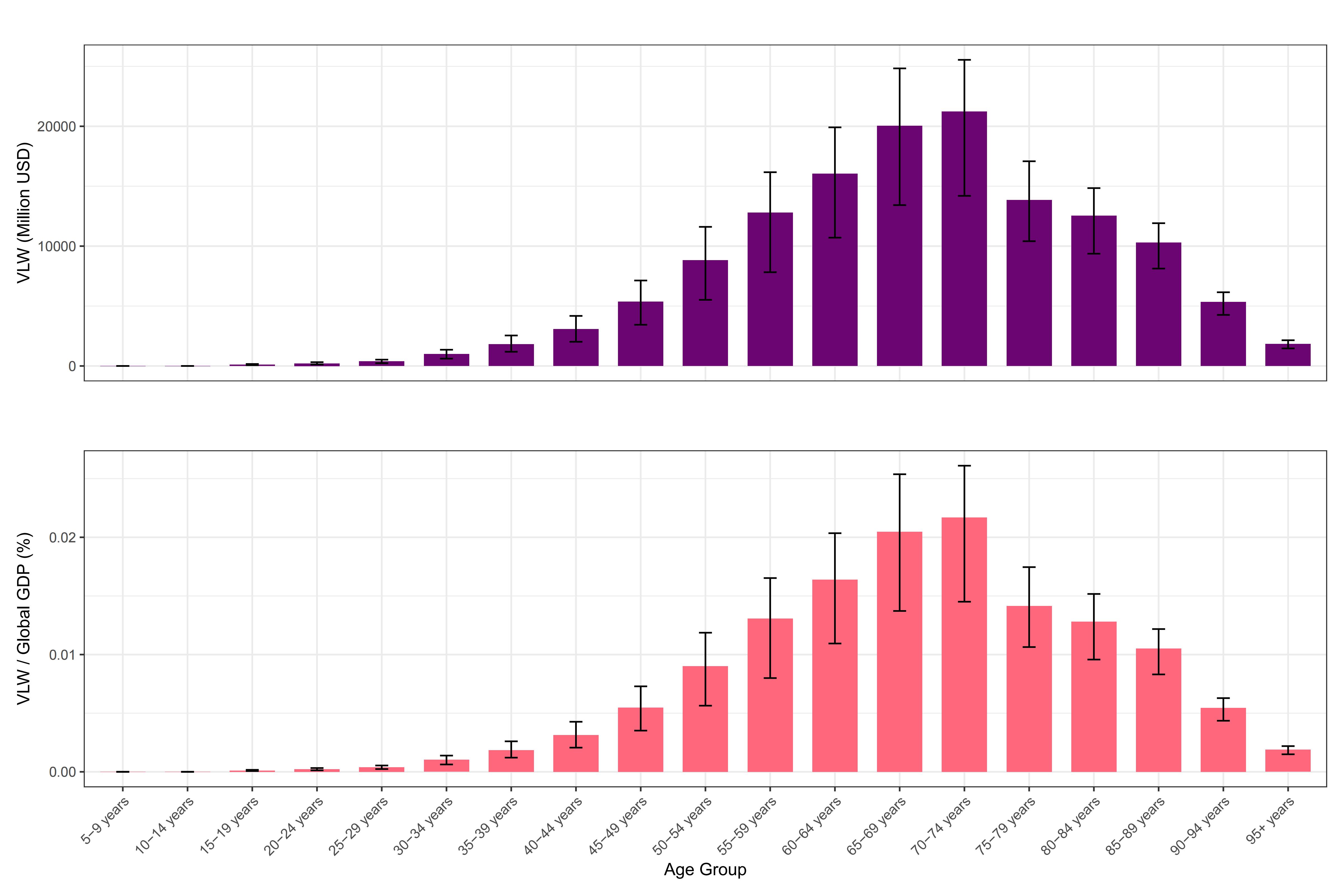


A

B

Supplementary Figure 5. VLW and VLW/GDP of different age groups for breast cancer in 2021, using IE at 1.55. A is man breast cancer; B is female breast cancer. VLW, Value of Lost Welfare; GDP：Gross Domestic Product

A


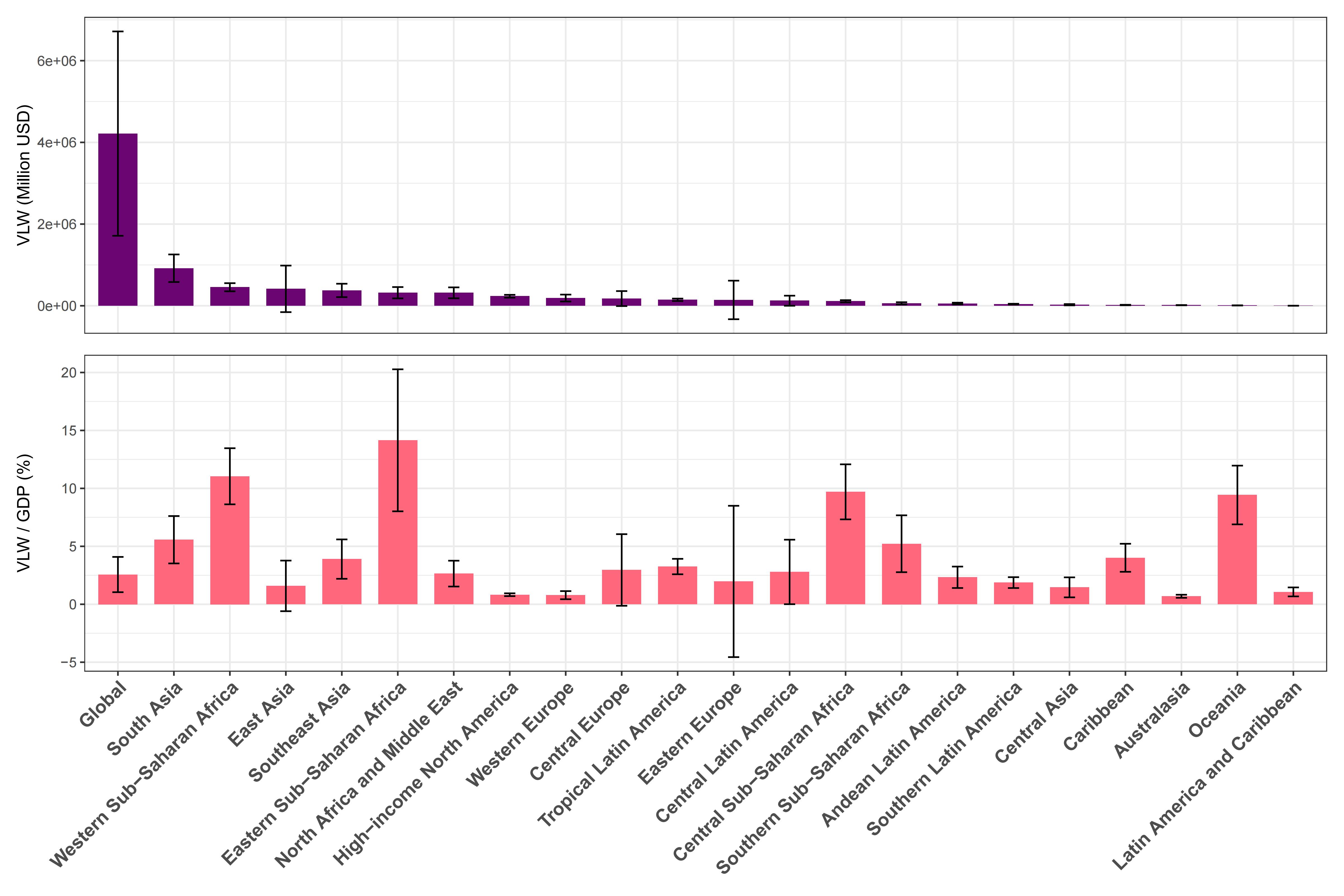

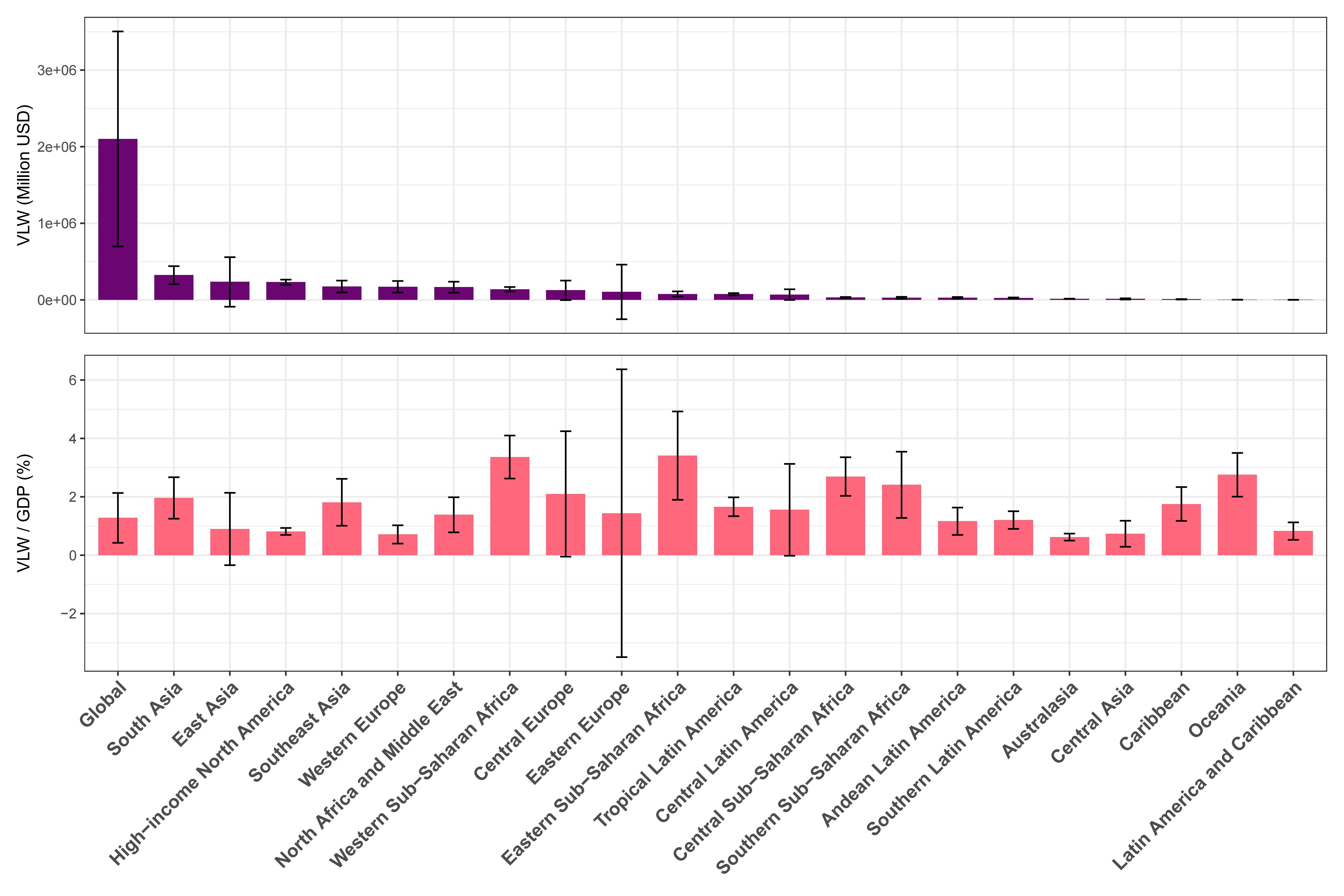

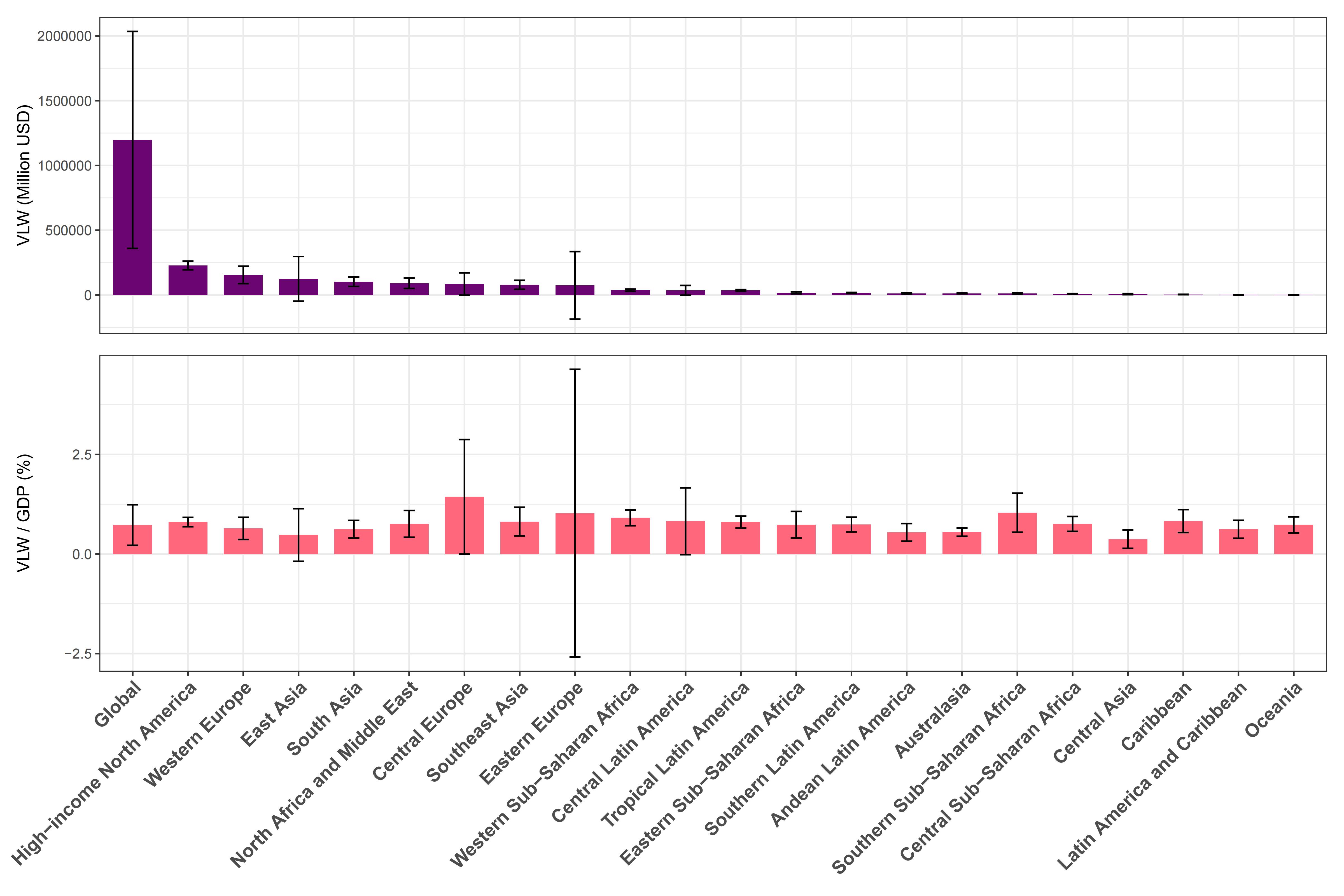


B

C

Supplementary Figure 6. VLW and VLW/GDP of global and regional prediction of breast cancer in 2050. A using IE at 0.55, B using IE at 1.0, C using IE at 1.5; VLW, Value of Lost Welfare; GDP, Gross Domestic Product; GBD,Global Burden of Disease; PPP, Purchasing Power Parity; IE, income elasticity


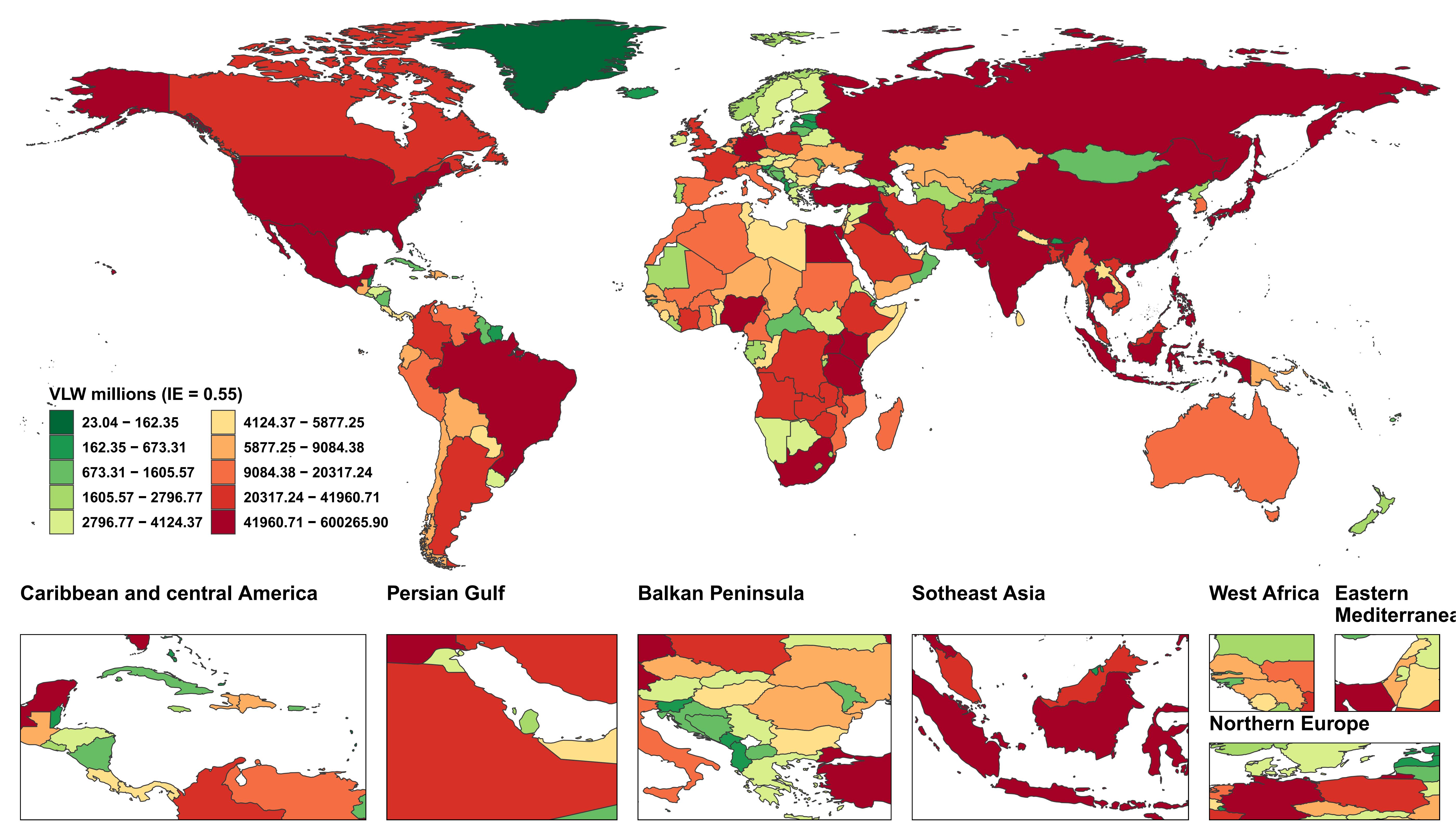

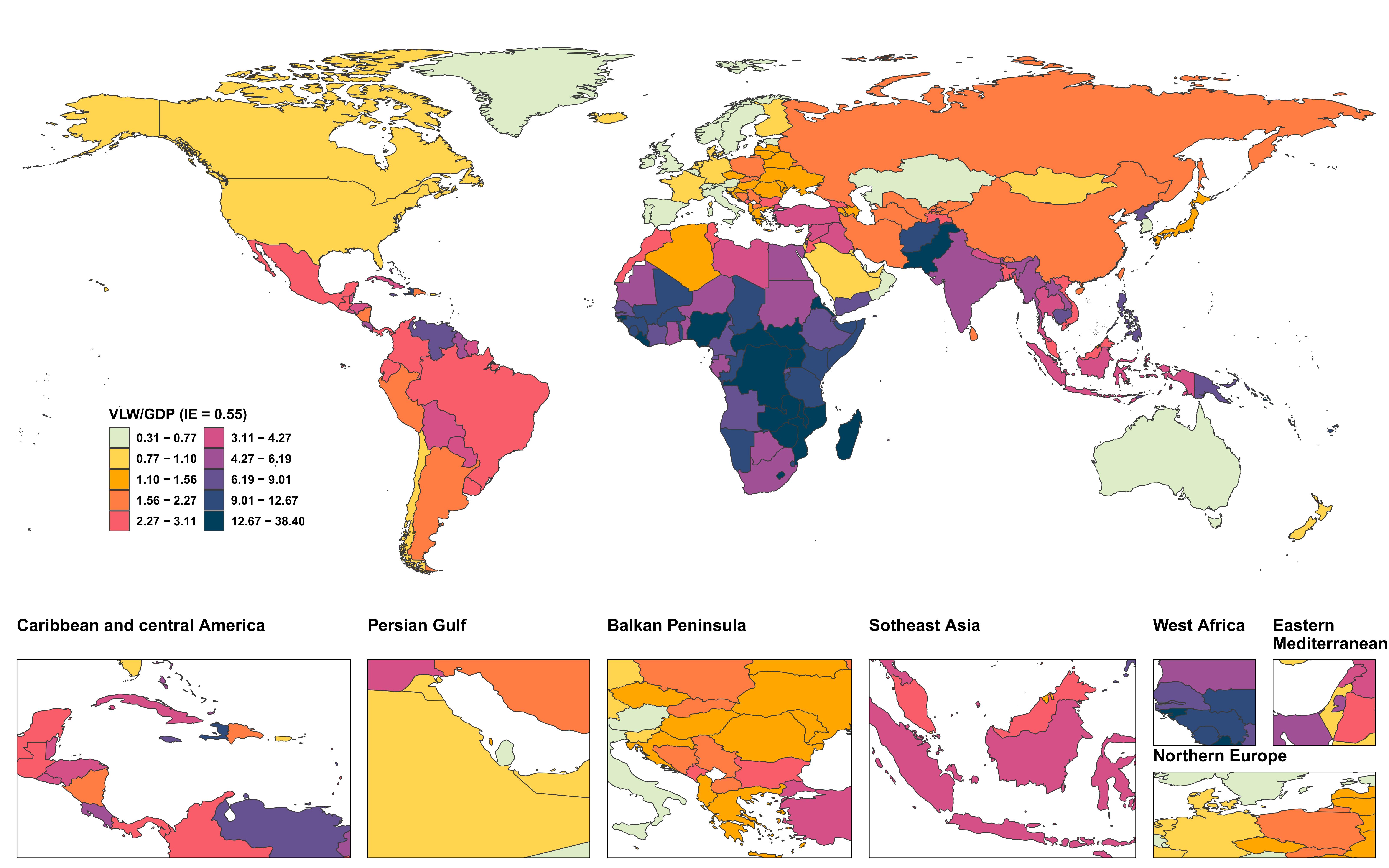


A

B

Supplementary Figure 7. World heat maps of VLW and VLW/GDP by country for breast cancer in 2050, using IE at 0.55. A is the world heat map of VLW; B is the world heat map of VLW. VLW, Value of Lost Welfare; GDP, Gross Domestic Product; GBD,Global Burden of Disease; PPP, Purchasing Power Parity; IE, income elasticity.


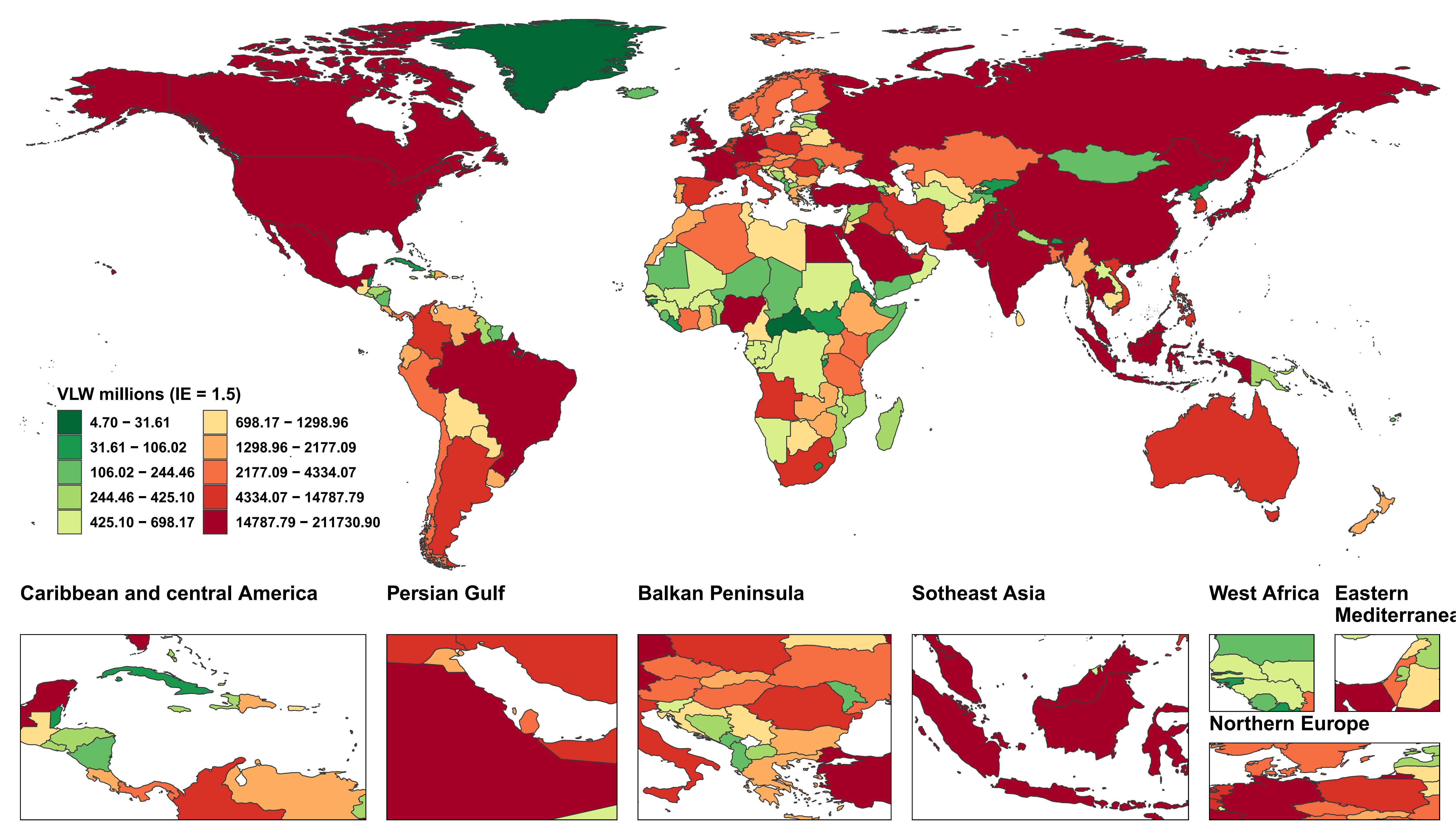


A


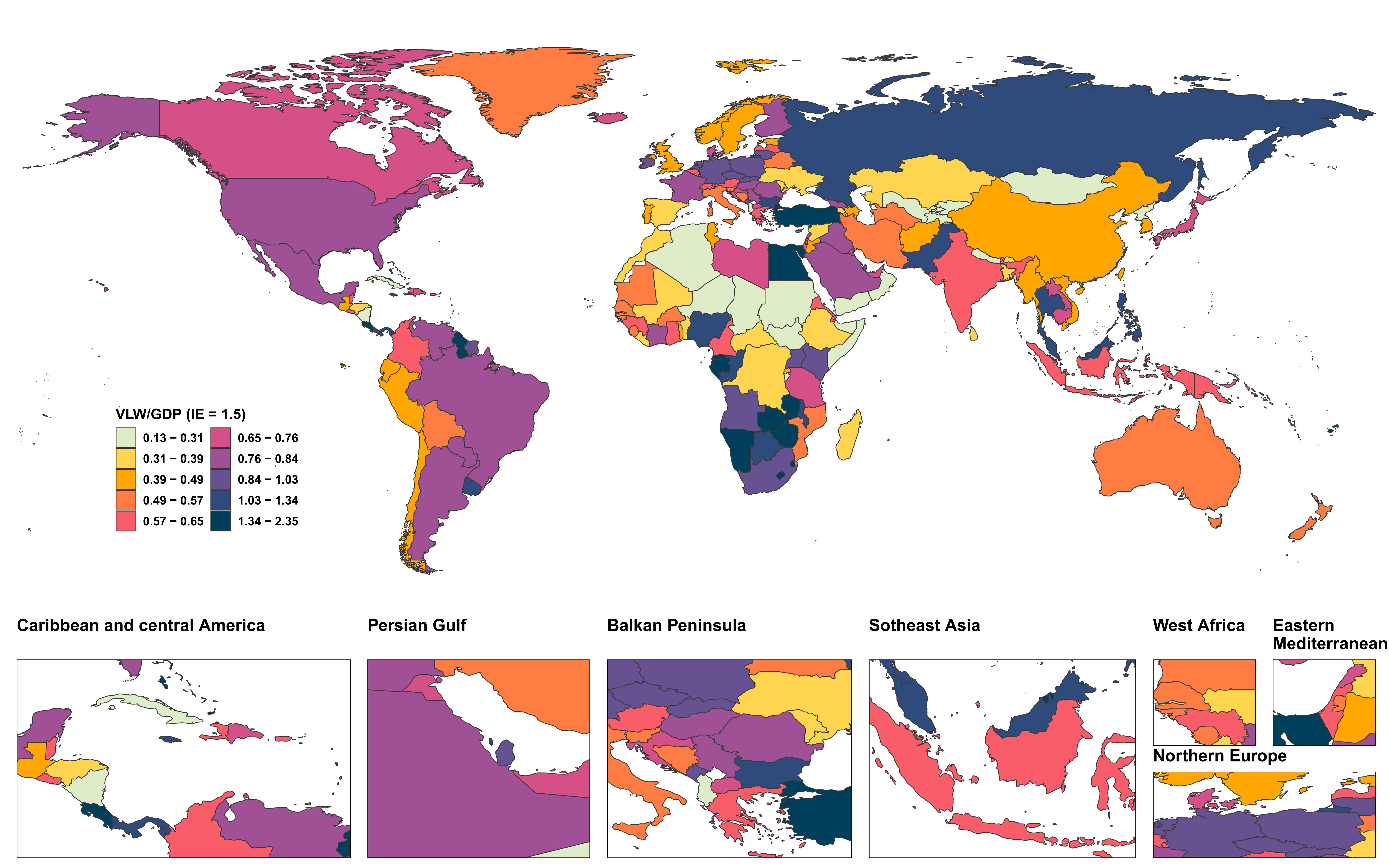


B

Supplementary Figure 8. World heat maps of VLW and VLW/GDP by country for breast cancer in 2050, using IE at 1.5. A is the world heat map of VLW; B is the world heat map of VLW. VLW, Value of Lost Welfare; GDP, Gross Domestic Product; GBD,Global Burden of Disease; PPP, Purchasing Power Parity; IE, income elasticity.
